# Ethnic inequities in routine childhood vaccinations in England 2006–2021: an observational cohort study using electronic health records

**DOI:** 10.1016/j.eclinm.2023.102281

**Published:** 2023-10-27

**Authors:** Claire X. Zhang, Clare Bankhead, Maria A. Quigley, Chun Hei Kwok, Claire Carson

**Affiliations:** a National Perinatal Epidemiology Unit, Nuffield Department of Population Health, University of Oxford, United Kingdom; b Nuffield Department of Primary Care Health Sciences, University of Oxford, United Kingdom; c Big Data Institute, Li Ka Shing Centre for Health Information and Discovery, University of Oxford, United Kingdom

**Keywords:** Ethnicity, Vaccination, Immunisation, Child

## Abstract

**Background:**

Population groups that are underserved by England's childhood vaccination programme must be identified to address the country's declining vaccination coverage. We examined routine childhood vaccination coverage in England by maternal ethnicity between 2006 and 2021.

**Methods:**

We created first, second and fifth birthday cohorts using mother-child linked electronic health records from the Clinical Practice Research Datalink (CPRD) Aurum. After validation against the UK Health Security Agency (UKHSA) and National Health Service England (NHSE) annual statistical reports, we described vaccination coverage for each vaccine by ethnicity and year. We used modified Poisson regression to analyse the effect of ethnicity on receiving the primary and full course of each vaccine.

**Findings:**

Up to 1,170,804 children born after 1 April 2006 were included in the first birthday cohort, reducing to 645,492 by the fifth birthday. Children were followed up until 31 March 2021 at the latest. Children born to mothers in 9 minority ethnic groups and those of unknown ethnicity had lower vaccination coverage (61.3–97.5%) than the White British group (79.9–97.8%) for all vaccines. Indian, Pakistani, Bangladeshi, Chinese, Any other Asian background, and White and Asian ethnic groups had similar vaccination coverage to the White British group (above 90% for most vaccines in most years). Inequities particularly affected the Caribbean group (e.g. 61% coverage for the 6/5/4-in-1 full course in 2020–21 by children's fifth birthday; RR 0.66, 95% CI 0.6–0.74 compared with the White British group) and Any other Black, African and Caribbean background (e.g. coverage 68% for the MMR primary course in 2020–21; RR 0.71, 95% CI 0.64–0.78). These inequities widened over the study period. For example, the absolute difference in coverage between the Caribbean and White British groups for the full course of MMR increased from 12% in 2011–12 to 22% in 2019–20. These inequities remained even after accounting for sociodemographic, maternal and birth related factors, and also widened from primary course to full course.

**Interpretation:**

Our findings suggest that urgent policy action is needed to address the ethnic inequities throughout England's routine childhood vaccination programme, which have been worsening over time.

**Funding:**

University of Oxford Clarendon Fund, St Cross College and Nuffield Department of Population Health.

**Research in context**

**Evidence before this study**

We conducted and published a scoping review to describe all quantitative studies on ethnic differences and inequities in paediatric healthcare utilisation in the United Kingdom (UK) 2001–2021, including preventive care services like routine childhood vaccination. We re-ran this search in Embase and Medline on 29 September 2023 for studies published up until this date using the following combination of terms and their synonyms: “ethnicity” AND “paediatric” AND “vaccination” AND “UK”. We identified 14 studies concerning routine childhood vaccinations with marked variability in their analytical designs, ethnicity classification systems, vaccines of interest, locations examined and time periods covered. The resultant mixed findings makes it challenging to synthesise and interpret the body of evidence to date.

**Added value of this study**

Previous research identified regional, city and borough level ethnic inequities for specific vaccines at various time points in the last two decades. Our longitudinal study builds upon this evidence by demonstrating that inequities span England's routine childhood vaccination schedule at a national level. Gaps between some minority ethnic groups and the White British group have widened over time, even prior to the COVID-19 pandemic. Additionally, being one of very few childhood vaccination studies to have used disaggregated ethnic categories, our findings have greater potential to informed tailored public health action by highlighting the significant heterogeneity within aggregated ethnic groups like ‘White’.

**Implications of all the available evidence**

The mounting concern for England's declining childhood vaccination coverage and growing risk of vaccine-preventable disease outbreaks and hospitalisations should be channelled into efforts to reduce the disproportionate impact on minority ethnic groups. Together with the existing qualitative evidence on challenges regarding vaccine confidence and access barriers, our findings highlight the urgent need for policy makers, commissioners and primary care services in England to co-produce solutions with minority ethnic communities and address inequities at national and local levels.

## Introduction

Routine childhood vaccination rates have become an increasing concern in England, with a declining trend in coverage over the past decade. In 2021–22, none of the vaccines in the national childhood vaccination programme met the World Health Organization (WHO) coverage target of 95%.^1^ To improve vaccination coverage and ensure equitable provision, the population groups that are underserved by the national programme must be identified.

The COVID-19 vaccination programme quickly gave rise to body of evidence on inequities in vaccine access and confidence, particularly highlighting inequities by ethnicity in England.^2^ COVID-19 vaccine coverage was poor for adults of Caribbean and African ethnicity, and vaccine confidence was also low in other minority ethnic groups compared to the ‘White British and Irish’ ethnic group.^2^, ^3^, ^4^

Despite the routine childhood vaccination programme in England having been established for much longer than its COVID-19 counterpart, studies on routine childhood vaccine coverage by ethnicity are relatively old and have yielded mixed results due to marked variability in analytical methods. All studies reported ethnic inequities for the measles, mumps and rubella (MMR), 5-in-1 (diphtheria, tetanus, pertussis, polio and haemophilius influenza type B) and rotavirus vaccines.^5^, ^6^, ^7^, ^8^, ^9^, ^10^, ^11^ However, the ethnic groups affected differed depending on how studies aggregated and classified ethnic groups, as well as which time periods, vaccines and geographies they captured.^12^ Some vaccines like meningococcal group B (MenB) have been overlooked altogether. Furthermore, parental/caregiver ethnicity has a greater influence on childhood vaccination than child ethnicity.^13^ However, only one study in the early 2000s has examined the effect of maternal ethnicity on childhood vaccinations.^14^

To address these limitations in the evidence to date, we examined the effect of maternal ethnicity on childhood vaccinations for children aged 5 and under in England between 2006 and 2021, for all vaccines in the routine schedule with the exception of influenza. We used mother-child linked electronic health records (EHRs) from primary and secondary care to answer the primary research question: What proportion of children born to mothers in each ethnic group were vaccinated as per the recommended schedule by their first, second and fifth birthdays? We also answered the secondary questions: What is the effect of maternal ethnicity on childhood vaccinations, and does this change across the study period?

## Methods

### Study design

We conducted an observational cohort study using individual-level data spanning the financial years 2006–07 through 2020–21. We follow the REporting of studies Conducted using Observational Routinely-collected health Data (RECORD) guidelines^15^ detailed in Appendix 1.

### Ethics

This study (protocol number 21_000716) was approved through the Clinical Practice Research Datalink (CPRD) Research Data Governance process. The data is provided by patients and collected by the NHS as part of their care and support, so individual consent was not required.

### Data sources

We used EHR data from the CPRD Aurum primary care database, May 2022 build. This included the Mother-Baby Link and Pregnancy Register provided by CPRD to link mother and child records in order to identify the study cohort and obtain pregnancy and birth related data. The CPRD Aurum database is one of the largest primary care databases in the UK, and is largely representative of the general population of England in terms of age, sex and deprivation.^16^ The May 2022 build covered 20% of the general population of the UK.^17^

Individual-level primary care data were also linked by CPRD to Hospital Episode Statistics (HES) Admitted Patient Care (APC), which we used to assign ethnicity, and the Office for National Statistics (ONS) Index of Multiple Deprivation (IMD) and Rural-Urban Classification (RUC) data, which we used to derive additional sociodemographic variables.^18^

### Study cohort

**Fig. 1:**
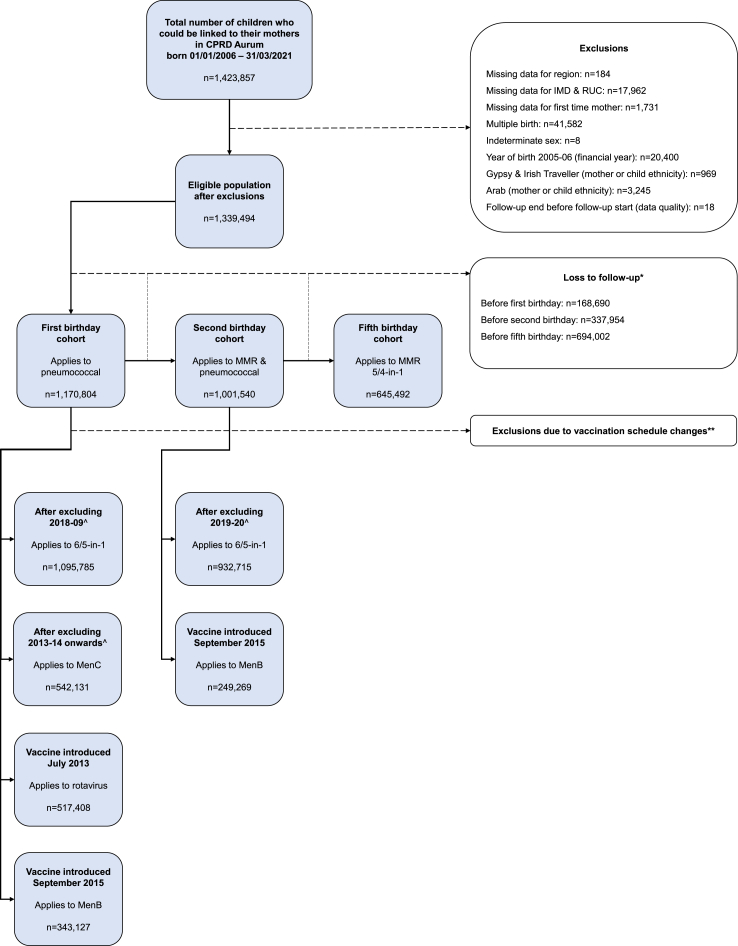
Study cohort inclusions and exclusions. ∗Loss to follow-up refers to children being excluded from one or more birthday cohorts if they died before reaching that birthday, the study period ended (31 March 2021), or they de-registered from their GP practice ∗∗Vaccination schedule changes are shown in Table 1. ˆ5-in-1 changed to 6-in-1 in 2018–19 for the first birthday cohort and 2019–20 for the second birthday cohort. Multiple vaccination schedule changes for MenC began in 2013–14 for the first birthday cohort. In these transition years, vaccine coverage in our cohort was more than 10% lower than UKHSA/NHSE annual statistical reports so we excluded these years from the analyses.

Children born after 1 January 2006 that could be linked to their mothers using the CPRD Aurum Mother-Baby Link were eligible for this study. As CPRD was the primary dataset used to derive key variables, linkage to HES and ONS was not a condition of cohort eligibility. We created three sub-cohorts (hereon referred to as ‘birthday cohorts’) by following children from birth until their first, second and fifth birthdays. Children were excluded from one or more of these birthday cohorts if they died before reaching that birthday, the study period ended (31 March 2021), or they de-registered from their GP practice. We also restricted the cohort by children's age at GP registration (before first birthday) to balance completeness of primary care records with data quality issues from retrospective clinical coding, and applied additional restrictions for certain vaccines due to national vaccination schedule related changes (Fig. 1, Table 1). Some exclusions were applied due to missing data or categorical variables with less than 5 children vaccinated (or not vaccinated) per category in each ethnic group. Before applying these exclusions, we explored whether data were missing completely at random by comparing those with missing and non-missing data by ethnic group and study outcomes. The resultant study period was 1 April 2006–31 March 2021.

**Table 1:** Routine childhood vaccination schedule in England and UKHSA/NHSE annual statistical reporting schedule.

Vaccine	Type	Schedule changes^a^	Expected age at administration	Course	Cumulative number of doses expected	First birthday	Second birthday	Fifth birthday
MMR (measles, mumps, rubella)	Combined	None	1 year	Primary course	1		✓	**ˆ**
			3 years and 4 months	Booster	2			**✓**
5-in-1 (diphtheria, tetanus, pertussis, polio, haemophilius influenzae type B)	Combined	Until Jul 2017	8, 12 and 16 weeks	Primary course	3	✓	ˆ	**ˆ**
6-in-1 (diphtheria, tetanus, pertussis, polio, haemophilius influenzae type B, hepatitis B)		From Aug 2017				✓	ˆ	
4-in-1 preschool booster (diptheria, tetanus, pertussis, polio)		None	3 years 4 months	Booster	4			**✓**
MenC (meningococcal group C)	Single	Until May 2013	12 and 16 weeks	Primary course	2	✓		
		Between Jun 2013 and Jun 2016	12 weeks		1	✓		
MenC and Hib (haemophilius influenzae type B)	Combined	None	1 year	Hib booster MenC booster until Jun 2016, primary course thereafter	1		✓	**ˆ**
MenB (meningococcal group B)	Single	From Sep 2015	8 and 16 weeks	Primary course	2	✓		
			1 year	Booster	3		✓	
Rotavirus	Single	From Jul 2013	8 and 12 weeks	Primary course	2	✓		
Pneumococcal	Single	Until Dec 2019	8 and 16 weeks	Primary course	2	✓		
			1 year	Booster	3		✓	
		From Jan 2020	12 weeks	Primary course	1	✓		
			1 year	Booster	2		✓	

✓Vaccine course expected to be completed by this birthday (used in descriptive and statistical analyses).

ˆRepeated measurement of a vaccine course at subsequent birthdays (only used to validate CPRD Aurum against the UKHSA/NHSE annual vaccination statistical reports).

a During the present study period 2006–2021.

### Deriving and validating vaccination outcomes

We identified vaccination by applying code lists (Appendix 2) to the Observations and Drug Issue (medical products) relational data tables in CPRD Aurum. We adapted these code lists from previously published lists and expanded on them using codes from the CPRD Aurum code browser version May 2022. For combined vaccines (MMR, 5-in-1, 6-in-1, 4-in-1 preschool booster and Hib/MenC), we developed an algorithm to assign the most plausible vaccine to codes that were ambiguous or only partially accounted for the possible vaccine(s) of interest. For example, a code for Hib vaccine could indicate that 6-in-1 or Hib/MenC vaccine was administered. This algorithm and further data cleaning processes are detailed in Appendix 2.

To align with the UK Heath Security Agency (UKHSA) and National Health Service England (NHSE) annual vaccination reports,^1^ we grouped children into the financial year in which they had their first, second and fifth birthdays. Financial years begin on 1 April and end 30 March. To validate the identification of routine childhood vaccinations in CPRD Aurum, we compared the annual proportions of children in our cohort who completed the primary course, or primary course plus booster (hereon referred to as ‘full course’), of each vaccine to that of the UKHSA/NHSE reports for the relevant birthday cohorts (Table 1, Appendix 3). For all subsequent analyses, we dropped data for vaccines in years where our cohort's vaccination rates were greater than or less than UKHSA/NHSE by 10% or more as we did not consider these years to be representative of vaccine coverage in the general population. These deviations are discussed in Appendix 3.

### Deriving ethnicity

We adopt the definition that ethnicity is a multidimensional and self-identified concept. It is socially constructed, defined by sociopolitical and geographic context, and evolves over time.^19^

We assigned maternal ethnicity by applying a code list adapted from previously published lists to the Observations data table in Aurum. Where individuals had missing records or ethnicity coded as ‘unknown’ or ‘not stated’ in Aurum, we used their HES APC episode-level ethnicity data. To assign a single ethnic category for individuals who had multiple ethnicity records, we adapted and used previously published algorithms for CPRD and HES to prioritise records by frequency, then recentness, then the ethnicity most commonly occurring in the general population.^20^^,^^21^ Additional methodology for assigning ethnicity is detailed in Appendix 4.

We then categorised individuals' final assigned ethnicity using the ONS England and Wales Census disaggregated ethnicity classification system. While we categorised ethnicity codes using the 2011 system, we did not examine the Gypsy and Irish Traveller and Arab ethnic groups (nor the Roma ethnic group from the 2021 Census) as their small group sizes would result in underpowered analysis, application of statistical process control to almost all outputs, and limit our ability to interpret findings meaningfully. Individuals of Gypsy, Roma, and Irish Traveller ethnicities registered at CPRD GP practices are also unlikely to be representative of their broader ethnic groups that experience significant challenges with GP registration.^22^ Our study therefore included 16 ethnic groups and an ‘Unknown’ group.

### Statistical analysis

**Fig. 2:**
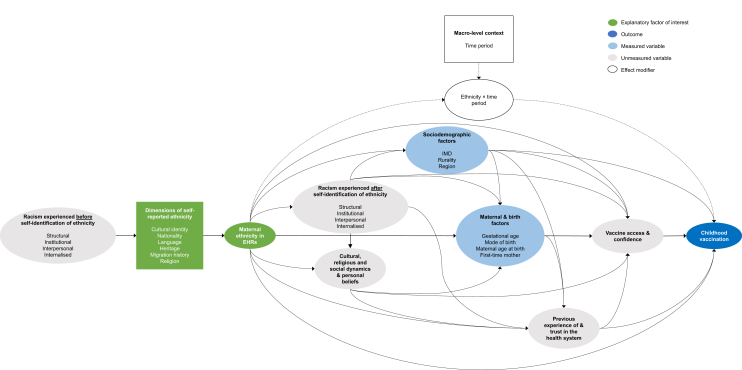
Directed acyclic graph (DAG) for the effect of maternal ethnicity on routine childhood vaccinations in England. Green nodes represent the explanatory factor of interest. Dark blue notes represent the outcome of interest. Light blue nodes represent measured variables, and grey nodes the unmeasured variables. The effect modifier (time period) has been represented shown in white.

We constructed a directed acyclic graph (DAG) informed by the existing evidence base to guide statistical modelling (Fig. 2).^23^, ^24^, ^25^, ^26^, ^27^, ^28^ In Appendix 5 we discuss the assumptions and simplifications made in the DAG, and detail the methods for deriving the measured variables in this DAG. As all measured and unmeasured variables in the DAG were ancestor variables of ethnicity or mediators, no variable adjustment was required for the main analysis.

In line with Table 1, we calculated the proportion of children born to mothers in each ethnic group that received the primary course or full course of each vaccine by the expected birthday (hereon referred to as vaccination ‘coverage’). We then stratified this by year of vaccination.

We examined the average (total) effect of maternal ethnicity on coverage of each course of each vaccine using risk ratios estimated from modified Poisson modelling with robust standard errors. We used likelihood ratio tests (LRT) to test the significance of effect modification by time period. We categorised year of vaccination into groups of three years with the exception of 2007–08 (where coverage was lower across multiple vaccines) and 2020–21 (COVID-19 pandemic). Where there was some evidence of effect modification (LRT p < 0.1), findings were reported stratified by time period, comparing each ethnic group to the English, Welsh, Scottish, Northern Irish or British group (hereon referred to as the White British group) in that same time period.

We could not measure various upstream mediators in our DAG using EHRs, and therefore could not conduct an unbiased formal mediation analysis for policy-relevant questions such as the relative contribution of sociodemographic, maternal and birth related factors to the effect of interest. Instead, we conducted an exploratory analysis of the effect of ethnicity on vaccinations after accounting for sociodemographic and maternal/birth related factors by through multivariable adjustment.

No statistical methods were needed to account for loss to follow-up as participant characteristics were not notably different when comparing the total eligible population after exclusions to each birthday cohort. We conducted all analyses in R (version ×64 4.1.2).

### Patient and Public Involvement (PPI)

We held a consultative online workshop with a PPI advisory group of five mothers/peer supporters from different ethnic backgrounds across different regions of England. We presented our findings to this advisory group and triangulated our evidence-informed interpretations with parents’ perspectives. We then collaboratively developed culturally-sensitive and parent-sensitive recommendations for policy, practice and future research.

### Role of the funding source

This study was funded by the University of Oxford Clarendon Fund, St Cross College and Nuffield Department of Population Health as part of a Doctor of Philosophy scholarship held by CZ. The funding source had no involvement in the study.

## Results

### Cohort characteristics

Of the 1,339,494 children eligible for this study, 99.6% of children and 99.2% of their mothers were linked to HES and ONS data. 1,170,804 (84%) were followed up until their first birthday, 1,001,540 (75%) until their second birthday and 645,492 (48%) until their fifth birthday. Maternal ethnicity was largely representative of the general population of women of child-bearing age (15–49 years) at the time of 2011 Census,^29^ as were most other sociodemographic, maternal and birth factors. There was a slightly lower proportion of children born preterm in our study cohort, and some regions over/underrepresented (Table 2).

**Table 2:** Characteristics of each birthday cohort compared to the eligible population after exclusions and the general population of England.

Participant characteristic	%	n (%)			
	National reference populations (see footnotes)	Eligible population after exclusions	First birthday cohort	Second birthday cohort	Fifth birthday cohort
Maternal ethnicity^a^					
English, Welsh, Scottish, Northern Irish or British	74.8	912,382 (68.9)	806,511 (69.6)	699,597 (70.6)	468,925 (73.5)
Irish	0.8	7093 (0.5)	6047 (0.5)	4998 (0.5)	2884 (0.5)
Any other White background	6.9	124,759 (9.4)	105,084 (9.1)	85,043 (8.6)	44,936 (7)
Indian	3.1	43,572 (3.3)	37,664 (3.3)	32,103 (3.2)	20,053 (3.1)
Pakistani	2.4	40,842 (3.1)	35,912 (3.1)	31,143 (3.1)	20,551 (3.2)
Bangladeshi	0.9	18,299 (1.4)	16,041 (1.4)	13,764 (1.4)	8752 (1.4)
Chinese	1.1	10,484 (0.8)	8933 (0.8)	7281 (0.7)	4089 (0.6)
Any other Asian background	2.1	33,495 (2.5)	28,722 (2.5)	24,014 (2.4)	14,468 (2.3)
Caribbean	1.3	13,695 (1)	11,893 (1)	9975 (1)	6095 (1)
African	2.5	56,682 (4.3)	47,823 (4.1)	39,165 (4)	22,754 (3.6)
Any other Black, African or Caribbean background	0.6	8858 (0.7)	7576 (0.7)	6364 (0.6)	3790 (0.6)
White and Black Caribbean	0.8	8931 (0.7)	7597 (0.7)	6263 (0.6)	3787 (0.6)
White and Black African	0.3	5231 (0.4)	4384 (0.4)	3529 (0.4)	1982 (0.3)
White and Asian	0.6	4374 (0.3)	3710 (0.3)	3079 (0.3)	1747 (0.3)
Any other Mixed or multiple ethnic background	0.6	8992 (0.7)	7474 (0.6)	6017 (0.6)	3235 (0.5)
Any other ethnic group	0.7	27,322 (2.1)	22,941 (2)	18,610 (1.9)	10,313 (1.6)
Unknown^g^	N/A	14,483 (1.1)	12,492 (1.1)	10,595 (1.1)	7131 (1.1)
Maternal and child ethnicity					
Same	N/A	1,104,100 (82.4)	970,927 (82.9)	836,527 (83.5)	550,217 (85.2)
Different	N/A	235,394 (17.6)	199,877 (17.1)	165,013 (16.5)	95,275 (14.8)
Sex^b^					
Male	51.2	686,689 (51.3)	600,017 (51.2)	512,811 (51.2)	330,547 (51.2)
Female	48.8	652,805 (48.7)	570,787 (48.8)	488,729 (48.8)	314,945 (48.8)
Region^b^					
London	17.8	304,872 (22.8)	258,657 (22.1)	212,200 (21.2)	119,743 (18.6)
East Midlands	8.1	23,408 (1.7)	20,587 (1.8)	17,792 (1.8)	11,757 (1.8)
East of England	10.9	59,763 (4.5)	53,193 (4.5)	46,135 (4.6)	31,356 (4.9)
North East	4.5	44,463 (3.3)	39,377 (3.4)	34,404 (3.4)	23,548 (3.6)
North West	13	228,560 (17.1)	202,865 (17.3)	177,311 (17.7)	121,512 (18.8)
South East	16.1	274,460 (20.5)	240,646 (20.6)	206,369 (20.6)	134,874 (20.9)
South West	8.9	158,218 (11.8)	138,775 (11.9)	119,287 (11.9)	78,066 (12.1)
West Midlands	10.7	205,041 (15.3)	180,603 (15.4)	156,467 (15.6)	103,598 (16)
Yorkshire and The Humber	9.9	40,709 (3)	36,101 (3.1)	31,575 (3.2)	21,038 (3.3)
IMD (patient level)^b^					
1 (least deprived)	16.8	252,281 (18.8)	225,540 (19.3)	198,161 (19.8)	135,744 (21)
2	17.3	245,731 (18.3)	216,892 (18.5)	187,058 (18.7)	123,315 (19.1)
3	18.6	252,003 (18.8)	219,205 (18.7)	186,517 (18.6)	118,493 (18.4)
4	21.6	286,275 (21.4)	246,742 (21.1)	207,510 (20.7)	128,008 (19.8)
5 (most deprived)	25.7	303,204 (22.6)	262,425 (22.4)	222,294 (22.2)	139,932 (21.7)
Rurality (patient level)^b^					
Urban	86	1,176,876 (87.9)	1,025,449 (87.6)	874,256 (87.3)	558,120 (86.5)
Rural	14	162,618 (12.1)	145,355 (12.4)	127,284 (12.7)	87,372 (13.5)
Birth weight^c^					
Very low (<1500 g)	1.2	6363 (0.5)	5497 (0.5)	4657 (0.5)	2933 (0.5)
Low (1500–2499 g)	5.9	47,580 (3.6)	41,236 (3.5)	34,953 (3.5)	21,698 (3.4)
Normal (2500–3999 g)	80.8	1,166,755 (87.1)	1,018,648 (87)	870,352 (86.9)	560,333 (86.8)
High (≥4000 g)	11.3	118,796 (8.9)	105,423 (9)	91,578 (9.1)	60,528 (9.4)
Gestational age^d^					
Term (37–42 weeks)	92.2	1,275,760 (95.2)	1,115,405 (95.3)	954,620 (95.3)	616,157 (95.5)
Preterm (23–36 weeks)	7.8	63,734 (4.8)	55,399 (4.7)	46,920 (4.7)	29,335 (4.5)
Mode of birth^c^					
Spontaneous vaginal	62	917,305 (68.5)	804,835 (68.7)	691,173 (69)	452,971 (70.2)
Other mode of birth	38	422,189 (31.5)	192,521 (29.8)	310,367 (31)	192,521 (29.8)
First time mother^e^					
No	56.1	834,041 (62.3)	737,461 (63)	639,294 (63.8)	423,357 (65.6)
Yes	43.9	505,453 (37.7)	433,343 (37)	362,246 (36.2)	222,135 (34.4)
Maternal age at delivery^d^					
Under 20	5	37,944 (2.8)	31,819 (2.7)	26,053 (2.6)	16,660 (2.6)
20–24	18.6	191,589 (14.3)	164,522 (14.1)	138,154 (13.8)	88,311 (13.7)
25–29	27.7	346,707 (25.9)	302,414 (25.8)	257,571 (25.7)	163,848 (25.4)
30–34	28.6	428,642 (32)	375,937 (32.1)	322,282 (32.2)	205,949 (31.9)
35–39	16	265,645 (19.8)	234,963 (20.1)	204,095 (20.4)	134,547 (20.8)
40 and older	4	68,967 (5.1)	61,149 (5.2)	53,385 (5.3)	36,177 (5.6)
Year of birth (financial year)^f^					
2006–07	6.5	92,868 (6.9)	85,282 (7.3)	77,529 (7.7)	63,278 (9.8)
2007–08	6.7	94,138 (7)	87,111 (7.4)	79,543 (7.9)	64,690 (10)
2008–09	6.8	96,503 (7.2)	89,006 (7.6)	80,662 (8.1)	65,342 (10.1)
2009–10	6.8	99,517 (7.4)	91,536 (7.8)	83,053 (8.3)	67,232 (10.4)
2010–11	7	102,555 (7.7)	94,165 (8)	85,234 (8.5)	68,575 (10.6)
2011–12	7	103,671 (7.7)	95,031 (8.1)	85,900 (8.6)	69,645 (10.8)
2012–13	7.1	98,744 (7.4)	90,524 (7.7)	81,856 (8.2)	66,712 (10.3)
2013–14	6.8	90,647 (6.8)	83,515 (7.1)	76,215 (7.6)	62,808 (9.7)
2014–15	6.7	84,604 (6.3)	78,520 (6.7)	71,990 (7.2)	59,164 (9.2)
2015–16	6.8	84,522 (6.3)	78,575 (6.7)	72,119 (7.2)	58,046 (9)
2016–17	6.7	83,573 (6.2)	77,890 (6.7)	71,501 (7.1)	N/A
2017–18	6.6	80,732 (6)	75,019 (6.4)	68,825 (6.9)	N/A
2018–19	6.4	79,632 (5.9)	73,708 (6.3)	67,113 (6.7)	N/A
2019–20	6.2	77,272 (5.8)	70,922 (6.1)	N/A	N/A
2020–21	6	70,516 (5.3)	85,282 (7.3)	N/A	N/A

Denominator is number of children.

a Reference population: Women aged 15–49 years in England at the time of the 2011 Census.

b Reference population: Children aged 0–4 years in England at the time of the 2011 Census.

c Reference population: Live births in England 2011.

d Reference population: Live births in England & Wales 2011.

e Reference population: National Maternity and Perinatal Audit in England 2017–18 using parity as a proxy.

f Reference population: Live births in England by calendar year 2006–2020.

g To ensure comparability with national reference data where ethnicity is known for all individuals, the denominator for known ethnic groups is the total number of children with known ethnicity. The denominator for the Unknown ethnic group is the total number of children.

### Childhood vaccination coverage by maternal ethnicity and year

While vaccination coverage varied between ethnic groups, patterns by ethnic group were similar across all vaccines and birthday cohorts. When all years were combined for the first birthday cohort, children born to mothers of White British ethnicity had coverage greater than or equal to the 95% target for the primary course of 6/5-in-1, MenB and pneumococcal. A similar trend was observed for children born to mothers of Indian, Chinese, Any other Asian background, and White and Asian ethnicities.

**Fig. 3:**
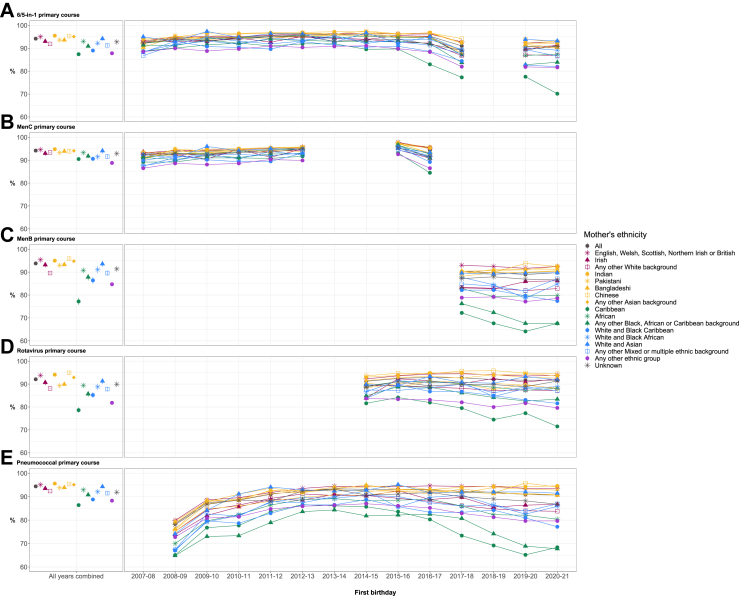
First birthday vaccination coverage by maternal ethnic group and year of vaccination. Vaccinations include the primary course of A. 6/5-in-1, B. MenC, C. MenB, D. Rotavirus, and E. Pneumococcal. Standard error bars are included for ‘all years combined’.

Children born to mothers from four different ethnic groups were much less likely to have received the primary course of any of the five vaccines due by this age: Caribbean, Any other ethnic group, White and Black Caribbean, and Any other Black, African or Caribbean background. Coverage in these four ethnic groups also began to decrease around 2014–15 to 2016–17 depending on the vaccine; while coverage remained above 90% for the White British ethnic group across the study period, by contrast these four ethnic groups dropped below 85% coverage for most vaccines by the first year of the COVID-19 pandemic (2020–21), with children of mothers of Caribbean ethnicity falling below 75% across all five vaccines (Fig. 3, with percentage coverage and absolute differences compared with the White British group detailed in Appendix 6).

**Fig. 4:**
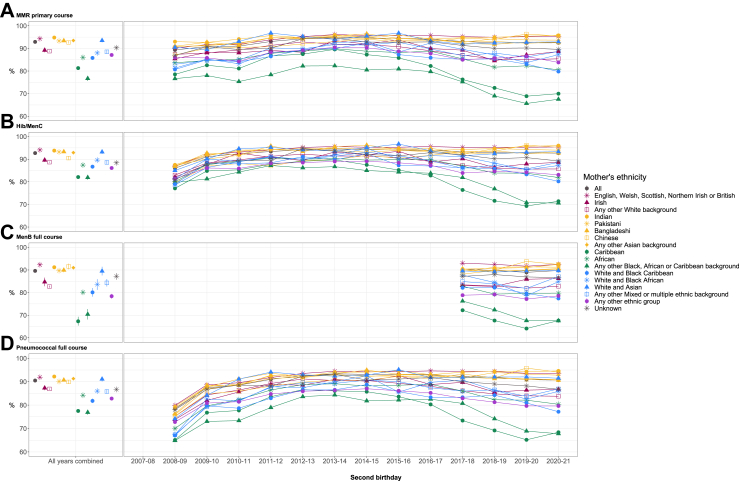
Second birthday vaccination coverage by maternal ethnic group and year of vaccination. Vaccinations include A. MMR primary course, B. Hib/MenC, C. MenB full course, and D. Pneumococcal full course. Standard error bars are included for ‘all years combined’.

A similar pattern by ethnic group was observed for the second birthday cohort (Fig. 4). The same ethnic groups as the first birthday cohort again had lower coverage than White British, and by children's second birthdays the gap was even wider between the White British group and the following groups: Irish, Any other White background, African, White and Black African, and Any other Mixed or multiple ethnic background. Most notably, children born to mothers from the Caribbean group and Any other Black, African or Caribbean background began with lower coverage than other groups in 2008–09, which fell to ≤70% for all vaccines by 2020–21, with the lowest being 64.1% for MenB in the Caribbean group.

**Fig. 5:**
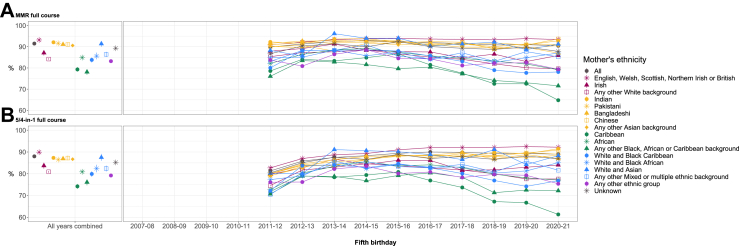
Fifth birthday vaccination coverage by maternal ethnic group and year of vaccination. Vaccinations include the full course of A. MMR, and B. 5/4-in-1. Standard error bars are included for ‘all years combined’.

The gap in coverage between the White British group and other ethnic groups persisted to children's fifth birthdays for the full course of MMR and 5/4-in-1. The overall coverage across all ethnic groups for the full course of these two vaccines was lower than the primary course, and the pattern by ethnic group remained the same (Fig. 5). Again, children born to mothers of Caribbean ethnicity had the lowest coverage, which fell over the latter half of the study period to a low of 61.3% in 2019–20 for the 5/4-in-1 vaccine. Fifth birthday coverage for Any other White background also dropped for these two vaccines by 2019–20 to approximately the same level as the White and Black Caribbean group and Any other ethnic group.

### Effect of maternal ethnicity on childhood vaccinations

In the main analysis, the effect of maternal ethnicity was observed across primary and full courses of all routine childhood vaccinations. For MMR, 6/5/4-in-1, Hib/MenC and pneumococcal, effect modification by time period was also observed, and ethnic inequities in vaccine coverage widened over the study period. There was no evidence that the effect of maternal ethnicity on vaccination for MenC, MenB and rotavirus differed over time; these were all vaccines that had been removed from or added to the national routine schedule during the study period, which limits the years of observation available.

**Fig. 6:**
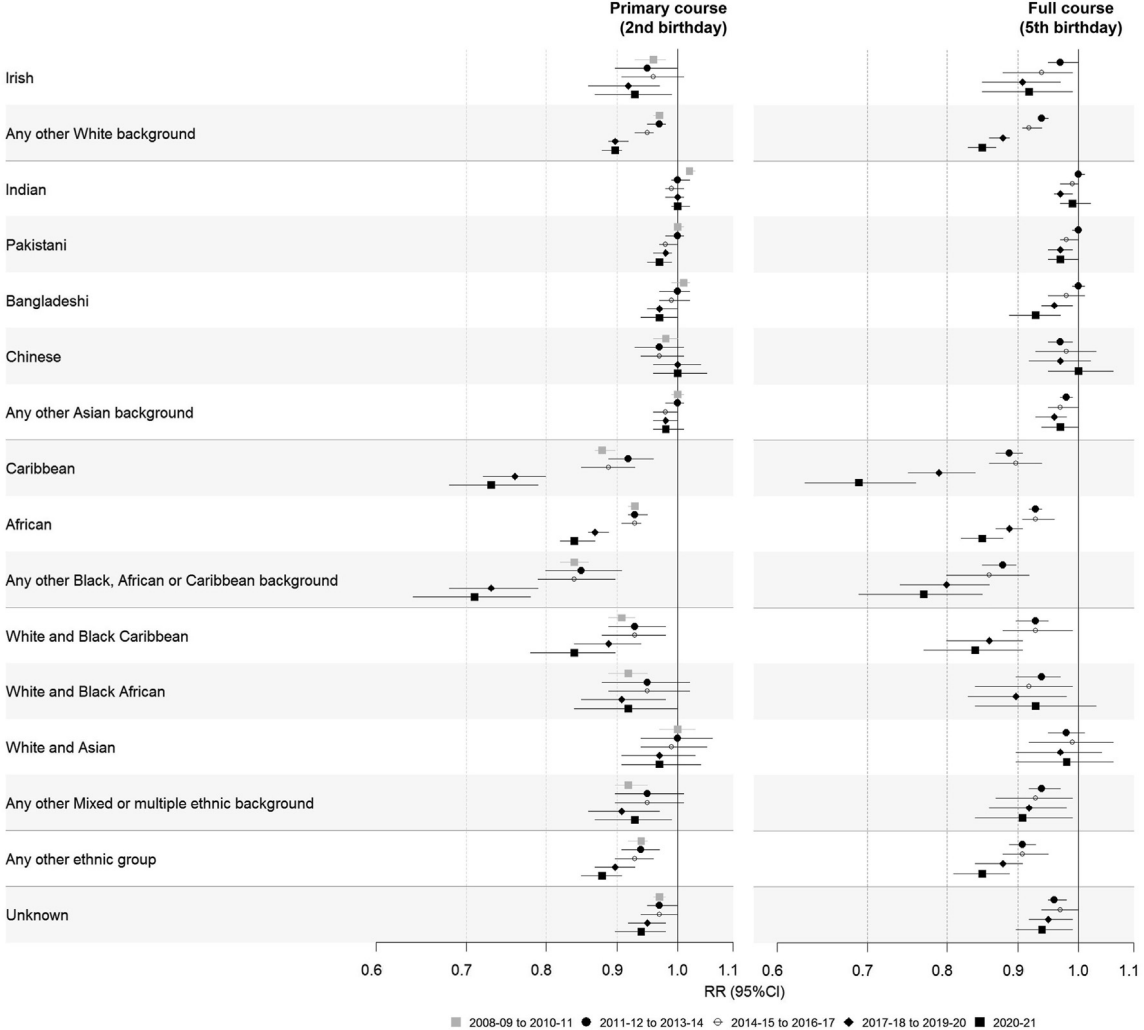
Average (total) effect of maternal ethnicity on MMR childhood vaccination, accounting for effect modification by time period (comparing each ethnic group with the White British reference group in the same time period). Coverage of the primary course is estimated for the second birthday cohort. Full course is estimated for the fifth birthday cohort.

Fig. 6 and Appendix 7 present the unadjusted risk ratios (RRs) for MMR within each stratum of time period, comparing each ethnic group with the White British group in the same time period. The effect of ethnicity on vaccination was strongest for two groups: Caribbean and Any other Black, African and Caribbean background. Children born to mothers of Caribbean ethnicity were 12% less likely to receive the primary course of MMR than the White British group for those who had their second birthday in 2007–08 (RR 0.88, 95% CI 0.87–0.9). This gap widened over the study period to 27% in 2020–21 (RR 0.73, 95% CI 0.68–0.79). The same was seen for the Any other Black, African and Caribbean background, where the likelihood of receiving the MMR primary course widened from 16% less likely compared to the White British group in 2007–08 (RR 0.84, 95% CI 0.82–0.86) to 29% less likely in 2020–21 (RR 0.71, 95% CI 0.64–0.78). Effect modification was also observed for the African ethnic group, Any other White background and Any other ethnic group, with RRs also increasing in magnitude over time albeit to a lesser extent than the other two minority ethnic groups. While effect modification was not observed for the White and Black Caribbean group and those with unknown maternal ethnicity, the likelihood of these groups receiving the MMR primary course was still lower than the White British group in almost all time periods.

For all vaccines, the gap in coverage between White British and most other minority ethnic groups was even wider for the full course than the primary course (Appendix 7). For example, for the 6/5/4-in-1 vaccine in 2020–21, the Caribbean group was 23% less likely to receive the primary course by the first birthday (RR 0.76, 95% CI 0.7–0.82) and 34% less likely to receive the full course by the fifth birthday (RR 0.66 95% CI 0.6–0.74).

In the secondary exploratory analysis, we observed minimal differences in effect estimates across all ethnic groups, time periods and vaccines after adjusting for sociodemographic, maternal and birth related factors (Appendix 8).

## Discussion

Between 2006 and 2021, children born to mothers in nine minority ethnic groups had lower vaccination coverage (range 61.3–97.5%) than the White British ethnic group (79.9–97.8%) for all vaccines in England's routine schedule. The range in coverage masks differences between ethnic groups and over time. Inequities in vaccine provision widened over the study period even before the COVID-19 pandemic, particularly affecting the Caribbean ethnic group and Any other Black, African and Caribbean background. For example, the absolute difference in coverage between the Caribbean and White British groups for the full course of MMR increased from 12% in 2011–12 to 22% in 2019–20, then even further to 29% by 2020–21. Additionally, ethnic inequities persisted, and in some cases, worsened between children receiving their primary vaccine course to receiving their full course. Accounting for the mediating effect of sociodemographic, maternal and birth related factors made minimal difference to these findings.

Our longitudinal findings corroborate previous evidence at regional and city levels that childhood vaccination coverage is lower in various minority ethnic groups^5^ except ‘Asian’ groups.^7^, ^8^, ^9^, ^10^ The ethnic groups known to be most affected by inequities in COVID-19 vaccination^2^ are also those affected by inequities in routine childhood vaccination. Our study suggests that the previously identified low coverage in the ‘White’ aggregated ethnic group may have been attributable to poor vaccine provision for Irish and Any other White background, rather than the White British group.^7^ This adds weight to existing recommendations to avoid aggregated ethnic groupings in research.^12^^,^^30^ Explanations for the widening of inequities over time, such as insurgence of vaccine misinformation and disinformation through increasing adoption of social media^31^ or the possible occurrence of a catalytic event affecting specific minority ethnic groups, should be explored further. Changes in the routine vaccination programme over time and universal or targeted strategies to encourage vaccination could have also had differential impacts on provision, access and vaccine confidence for different ethnic groups.

The mounting concern for England's overall declining trend in childhood vaccination and increasing risk of vaccine-preventable disease outbreaks should be channeled into efforts to reduce the disproportionate impact on minority ethnic groups, especially since there may be varied service provision in areas with greater ethnic diversity. Existing qualitative evidence on the compounding factors that drive ethnic inequities in childhood vaccination^13^ and learnings from the COVID-19 vaccine programme could be used to develop top-down and bottom-up strategies for childhood vaccination.^32^ Given the long history of structural racism and intergenerational mistrust of health systems that particularly affect ethnic groups like the Caribbean community and other Black communities in England,^31^ rebuilding trusted information channels and creating safe forums for parents and caregivers to voice their concerns should be at the core of all vaccination equity strategies.^32^ Early infancy is also an immensely stressful period for parents, and the higher likelihood of poor birth experiences for minority ethnic mothers, particularly in Black ethnic groups,^33^ could have flow-on effects to decision-making during this period. As such, midwifery, health visiting and peer support programs tailored to the needs of mothers in the most underserved ethnic groups could empower parents and caregivers to make informed vaccination decisions prior to birth.^34^ Providing culturally and linguistically appropriate public health messages about the importance of vaccination for the health of children and their wider communities could also improve vaccine access and confidence.^13^

To achieve this, national level directives could be put in place to support GP practices, integrated care systems and local public health teams. Participatory action to understand specific challenges and concerns at a local level could be followed by co-production of vaccination strategies with minority ethnic communities.^32^ To evaluate and continually adapt these policy responses, policy makers and commissioners could consider using existing available ethnicity data to monitor vaccination by ethnicity in their local areas. This could be paired with implementation research to understand what strategies are effective, where, and for whom. Developing standards for consistent coding of both ethnicity and vaccinations in EHRs could assist with this and reduce the need for algorithmic determination of these variables. Vaccinations provided by other GP practices or in other countries should also be consistently coded in EHRs retrospectively to ensure accurate determination of vaccination status.

To our knowledge, this is the first study in England to develop and validate an algorithm in order to study the full schedule of childhood vaccinations using the CPRD Aurum database. Using CPRD Aurum to do this has enabled a large enough cohort size to examine vaccination coverage over time, and also allowed sufficient power to examine ethnicity at a disaggregated level. Given that parental ethnicity is more likely than child ethnicity to shape healthcare utilisation in infancy and early childhood,^13^ linking mother and child EHRs to examine maternal ethnicity as the explanatory factor of interest also increases the potential to inform tailored public health action. Paternal ethnicity is not available in CPRD-HES linked datasets. Future studies could explore the feasibility of father-child record linkages to facilitate studies of maternal, paternal and parental ethnicity as a complete unit and its effect on childhood vaccination and other health outcomes.

Using data from EHRs rather than vaccination-specific information management systems also means that, despite validation against the UKHSA/NHSE annual statistical reports, our vaccine coverage estimates will not exactly reflect true coverage in the general population. The study is also England-centric, so coverage estimates may not be directly comparable with international recommendations for timing of vaccine doses. Additionally, limiting cohort eligibility to mother–baby pairs who could be linked through CPRD's Mother-Baby Link algorithm likely resulted in inclusion of children with better data quality and potentially better engagement with primary care than those whose records could not be linked. While the measured characteristics of children were similar in all birthday cohorts, selection bias may have been introduced through differences in unmeasured characteristics such as family stability or social networks.

The proportion of children in the general population who are not registered with a GP practice is under 1%.^35^ Our dataset does not represent these children, that is, those who are the least likely to receive routine vaccinations, and as such we are unable to examine the ethnic distribution of this group nor its effect on childhood vaccination coverage. Furthermore, London is overrepresented in CPRD Aurum while Yorkshire and The Humber and regions in the East are underrepresented. This, together with insufficient group sizes for some ethnic groups in these smaller regions, meant that we could not study regional differences in vaccination coverage by ethnicity. Future research could fill these gaps by using alternate data sources to study coverage by region, and to examine coverage in Gypsy, Irish Traveller, Roma and Arab ethnic groups. The consequences of ethnic inequities in vaccination coverage should also be explored in future, such as inequities in vaccine-preventable disease prevalence, outbreaks and hospitalisations. Disease cases and hospitalisations prevented by redressing ethnic inequities in vaccination could also be quantified.

Ethnic inequities are persistent and pervasive across England's routine childhood vaccination programme at a national level, and have worsened over time for some minority ethnic groups. Our findings add quantitative weight to the existing qualitative evidence on barriers to vaccine confidence and access in minority ethnic groups. It highlights the urgent need for policy makers, commissioners and primary care services in England to prioritise public health resources and work with minority ethnic communities to understand parental concerns about vaccination and address inequities in provision and access.

## Contributors

CZ conceptualised and designed the study, and wrote the manuscript with input from all authors. CZ conducted data cleaning with the support of CHK. CZ also conducted data analysis with methodological input from CC, MQ and CB. All authors had full access to all the data in the study and had final responsibility for the decision to submit for publication. CHK and CZ have verified the underlying data.

## Data sharing statement

This study (protocol number 21_000716) was approved through the Clinical Practice Research Datalink (CPRD) Research Data Governance process. All data used in this study were provided by the CPRD, and cannot be shared with other researchers. Researchers must seek approval from CPRD to access this data for the purposes of their own research. This study is based in part on data from the CPRD obtained under licence from the UK Medicines and Healthcare products Regulatory Agency. The data is provided by patients and collected by the NHS as part of their care and support. The interpretation and conclusions contained in this study are those of the authors alone. Linked data were also provided by the Office for National Statistics (ONS). ONS and Hospital Episode Statistics (HES) data © (2021) were re-used with the permission of The Health & Social Care Information Centre. All rights reserved.

## Declaration of interests

This study was funded by the University of Oxford Clarendon Fund, St Cross College and Nuffield Department of Population Health as part of a Doctor of Philosophy scholarship held by CZ.
