# Supplementary material for: Ethnic inequities in routine childhood vaccinations in England 2006–2021: an observational cohort study using electronic health records
Source: eClinicalMedicine. 2023 Oct 27;65:102281. doi: 10.1016/j.eclinm.2023.102281 (PMC10641103; doi:10.1016/j.eclinm.2023.102281)
Supplement: Supplementary Appendix [file mmc1.pdf]

Supplementary appendix

Appendix 1. RECORD checklist for cohort studies 2
Appendix 2. Algorithm for deriving combined vaccines and further data cleaning processes 7
Appendix 3. Annual vaccination coverage in the study cohort compared with the UKHSA/NHS Digital annual routine statistical reports 9
Appendix 4. Algorithm for assigning the most plausible ethnicity & additional methodology for deriving ethnicity 15
Appendix 5. DAG construction and deriving other study variables 17
Appendix 6. Childhood vaccination coverage by ethnicity and financial year of vaccination, and absolute difference comparing each ethnic group with White British 19
Appendix 7. Main analysis: Average (total) effect of maternal ethnicity on MMR childhood vaccination, accounting for effect modification by time period where statistically significant (comparing each ethnic group with the White British reference group in the same time period) 30
Appendix 8. Exploratory analysis: Effect of maternal ethnicity on childhood vaccination, after adjustment for sociodemographic, maternal and birth related factors, and accounting for effect modification by time period where statistically significant (comparing each ethnic group with the White British reference group in the same time period) 36
References 42

Appendix 1. RECORD checklist for cohort studies

	Item No.	STROBE items	Location in manuscript where items are reported	RECORD items	Location in manuscript where items are reported
**Title and abstract**					
	1	(a) Indicate the study's design with a commonly used term in the title or the abstract (b) Provide in the abstract an informative and balanced summary of what was done and what was found	Title page, abstract p.1-2	RECORD 1·1: The type of data used should be specified in the title or abstract. When possible, the name of the databases used should be included.<br><br>RECORD 1·2: If applicable, the geographic region and timeframe within which the study took place should be reported in the title or abstract.<br><br>RECORD 1·3: If linkage between databases was conducted for the study, this should be clearly stated in the title or abstract.	Title page, abstract p.1-2
**Introduction**					
Background rationale	2	Explain the scientific background and rationale for the investigation being reported	Background p.4		
Objectives	3	State specific objectives, including any prespecified hypotheses	Background p.4		
**Methods**					
Study Design	4	Present key elements of study design early in the paper	Methods p.4		
Setting	5	Describe the setting, locations, and relevant dates, including periods of recruitment, exposure, follow-up, and data collection	Methods p.4-6		
Participants	6	*(a) Cohort study* - Give the eligibility criteria, and the sources and methods of selection of participants. Describe methods of follow-up<br>*Case-control study* - Give the eligibility criteria, and the sources and	Methods p.4-6	RECORD 6·1: The methods of study population selection (such as codes or algorithms used to identify subjects) should be listed in detail. If this is not possible, an explanation should be provided.	Appendix 2-4

		methods of case ascertainment and control selection. Give the rationale for the choice of cases and controls<br>*Cross-sectional study* - Give the eligibility criteria, and the sources and methods of selection of participants<br><br>*(b) Cohort study* - For matched studies, give matching criteria and number of exposed and unexposed<br>*Case-control study* - For matched studies, give matching criteria and the number of controls per case		RECORD 6·2: Any validation studies of the codes or algorithms used to select the population should be referenced. If validation was conducted for this study and not published elsewhere, detailed methods and results should be provided.<br><br>RECORD 6·3: If the study involved linkage of databases, consider use of a flow diagram or other graphical display to demonstrate the data linkage process, including the number of individuals with linked data at each stage.	
Variables	7	Clearly define all outcomes, exposures, predictors, potential confounders, and effect modifiers. Give diagnostic criteria, if applicable.	N/A	RECORD 7·1: A complete list of codes and algorithms used to classify exposures, outcomes, confounders, and effect modifiers should be provided. If these cannot be reported, an explanation should be provided.	Methods p.7-9, Appendix 2, 4, 5, GitHub
Data sources/ measurement	8	For each variable of interest, give sources of data and details of methods of assessment (measurement). Describe comparability of assessment methods if there is more than one group	Methods p.7-9		
Bias	9	Describe any efforts to address potential sources of bias	Methods p.4-5, 7<br>Appendix 2-5		
Study size	10	Explain how the study size was arrived at	Methods p.6		
Quantitative variables	11	Explain how quantitative variables were handled in the analyses. If applicable, describe which groupings were chosen, and why	Methods p.7-9, Appendix 5		
Statistical methods	12	(a) Describe all statistical methods, including those used to control for confounding<br>(b) Describe any methods used to examine subgroups and interactions	Methods p.4-9		

		(c) Explain how missing data were addressed<br>(d) *Cohort study* - If applicable, explain how loss to follow-up was addressed<br>*Case-control study* - If applicable, explain how matching of cases and controls was addressed<br>*Cross-sectional study* - If applicable, describe analytical methods taking account of sampling strategy<br>(e) Describe any sensitivity analyses			
Data access and cleaning methods		..		RECORD 12·1: Authors should describe the extent to which the investigators had access to the database population used to create the study population.<br><br>RECORD 12·2: Authors should provide information on the data cleaning methods used in the study.	Contributors p.22, Methods p.7-9, Appendix 2, 4, 5
Linkage		..		RECORD 12·3: State whether the study included person-level, institutional-level, or other data linkage across two or more databases. The methods of linkage and methods of linkage quality evaluation should be provided.	Methods p.4
**Results**					
Participants	13	(a) Report the numbers of individuals at each stage of the study (*e.g.*, numbers potentially eligible, examined for eligibility, confirmed eligible, included in the study, completing follow-up, and analysed)<br>(b) Give reasons for non-participation at each stage.<br>(c) Consider use of a flow diagram	Methods p.6, Results p.11-12	RECORD 13·1: Describe in detail the selection of the persons included in the study (*i.e.*, study population selection) including filtering based on data quality, data availability and linkage. The selection of included persons can be described in the text and/or by means of the study flow diagram.	Results p.11-12
Descriptive data	14	(a) Give characteristics of study participants (*e.g.*, demographic,	Results p.11-13		

		clinical, social) and information on exposures and potential confounders<br>(b) Indicate the number of participants with missing data for each variable of interest<br>(c) *Cohort study* - summarise follow-up time (*e.g.*, average and total amount)			
Outcome data	15	*Cohort study* - Report numbers of outcome events or summary measures over time<br>*Case-control study* - Report numbers in each exposure category, or summary measures of exposure<br>*Cross-sectional study* - Report numbers of outcome events or summary measures	Results p.14-17, Appendix 6		
Main results	16	(a) Give unadjusted estimates and, if applicable, confounder-adjusted estimates and their precision (e.g., 95% confidence interval). Make clear which confounders were adjusted for and why they were included<br>(b) Report category boundaries when continuous variables were categorized<br>(c) If relevant, consider translating estimates of relative risk into absolute risk for a meaningful time period	Results p.17-18, Appendix 7		
Other analyses	17	Report other analyses done—e.g., analyses of subgroups and interactions, and sensitivity analyses	Appendix 8		
**Discussion**					
Key results	18	Summarise key results with reference to study objectives	Discussion p.20		
Limitations	19	Discuss limitations of the study, taking into account sources of potential bias or imprecision. Discuss	Discussion p.20-21	RECORD 19·1: Discuss the implications of using data that were not created or collected to answer the specific research question(s). Include discussion of misclassification bias,	Discussion p.21

		both direction and magnitude of any potential bias		unmeasured confounding, missing data, and changing eligibility over time, as they pertain to the study being reported.	
Interpretation	20	Give a cautious overall interpretation of results considering objectives, limitations, multiplicity of analyses, results from similar studies, and other relevant evidence	Discussion p.20, Conclusion p.21		
Generalisability	21	Discuss the generalisability (external validity) of the study results	Discussion p.20-21		
**Other Information**					
Funding	22	Give the source of funding and the role of the funders for the present study and, if applicable, for the original study on which the present article is based	Methods p.11, Funding p.22		
Accessibility of protocol, raw data, and programming code		..		RECORD 22·1: Authors should provide information on how to access any supplemental information such as the study protocol, raw data, or programming code.	Appendix 2, 4, GitHub

Appendix 2. Algorithm for deriving combined vaccines and further data cleaning processes

The code lists and R scripts that we used to identify vaccines in CPRD Aurum have been made available in GitHub. We adapted code lists from previously published lists (1-3).

We excluded vaccination records if they were missing a date, occurred before the child's date of birth, or after the child's follow-up ended for any reason (fifth birthday, death, de-registration or end of the study period). For combined vaccines (MMR, 5-in-1, 6-in-1, preschool booster and Hib/MenC), we developed an algorithm (Supplemental figure 1) to assign the most plausible vaccine to codes that only partially accounted for the possible vaccine(s) of interest (e.g. a code for Hib vaccine could indicate that 6-in-1 or Hib/MenC vaccine was administered).

We then de-duplicated records for the same child and vaccine entered on the same date. For combined vaccines, where a child had both 'full' (e.g. MMR) and 'partial' codes (e.g. measles) recorded on the same date, and/or had 'medcodes' and 'prodcodes' recorded on the same date, we prioritised full over partial and 'medcode' over 'prodcode'. This resulted in one unique record of one dose per child per vaccine per date, enabling us to count the number doses of doses administered for each child. For each birthday cohort, only codes recorded before a child reached that particular birthday was counted.

We then used England's vaccine schedule (Table 1) to determine how many doses of which vaccine should have been administered by each child's first, second and fifth birthdays, and therefore whether they had completed their primary course or primary course plus booster for that vaccine by the expected birthday.

Table 1 also outlines when and how England's vaccine schedule changed over the years. We also accounted for these changes by setting birth date parameters when deriving sub-cohorts for some of the vaccines (Figure 1), and for determining whether the primary or booster dose had been completed for some vaccines. For example, for rotavirus, only those born after the vaccine was introduced were included in the rotavirus sub-cohorts. Similarly, for pneumococcal, children born after the schedule change for pneumococcal were only expected to have 1 dose for their primary course instead of 2.

Supplemental figure 1. Algorithm for assigning the most plausible combined vaccine using component codes

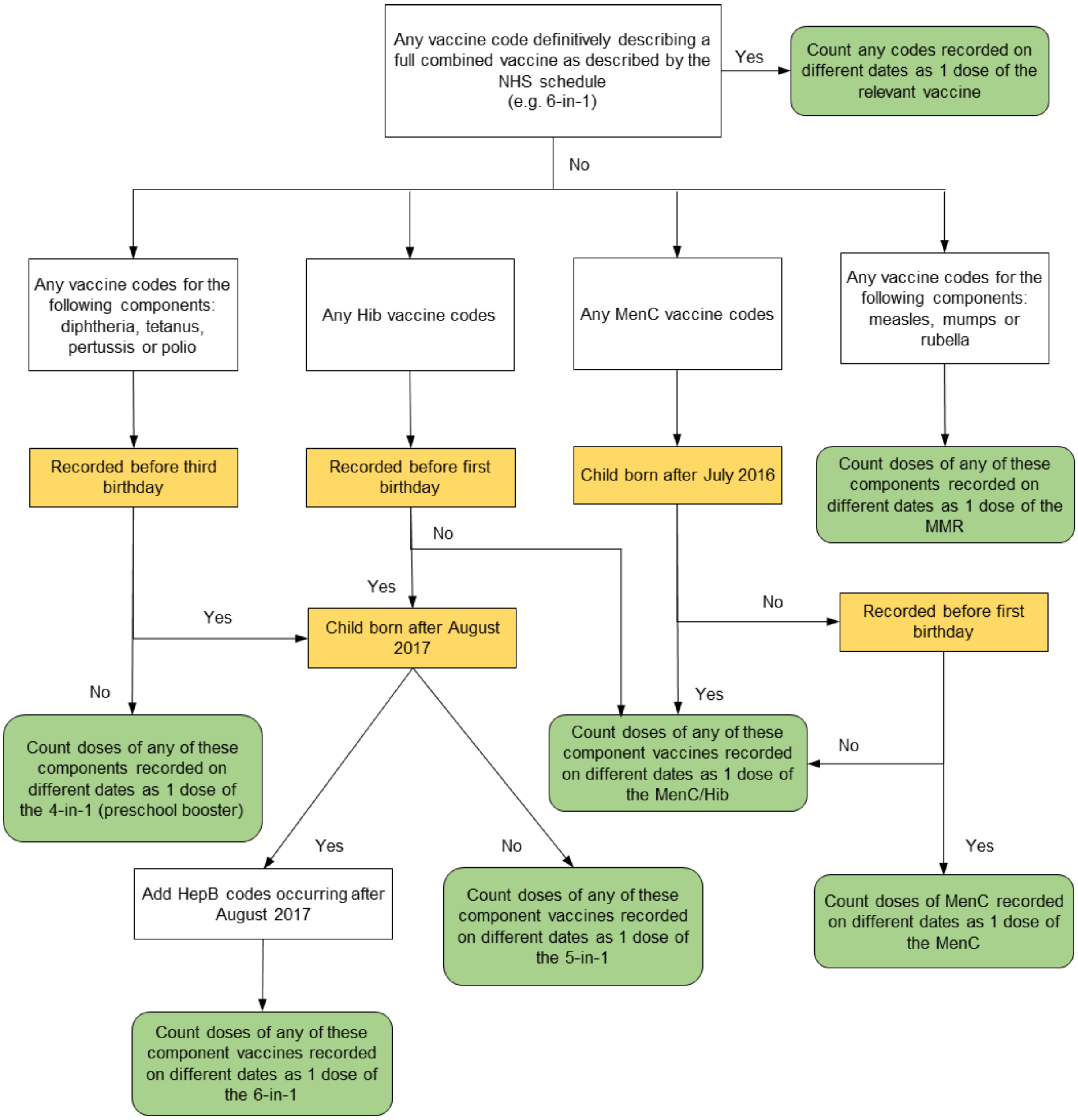

Appendix 3. Annual vaccination coverage in the study cohort compared with the UKHSA/NHS Digital annual routine statistical reports

Comparisons can only be made for the years, vaccines and birthday cohorts in which there is available data from both UKHSA/NHS Digital and our CPRD Aurum cohort. Reasons for data unavailability included:

- the children in our cohort couldn't have aged to a certain age in a given year (e.g. the first group born at the start of the study period could only reach their fifth birthday in 2011)
- some vaccines were only introduced part way into our study period
- UKHSA/NHS Digital did not collect data for every vaccine for every birthday cohort each year, i.e. there were gaps in their data due to issues with data collection and changes in the vaccine schedule

Boxes coloured in red indicate that the coverage in our cohort was greater than or less than UKHSA/NHS Digital by 10% or more. We suspect that deviations were due to national vaccination schedule changes. MenC schedule changes could have resulted in inconsistent clinical coding. 6/5/4-in-1 schedule changes could have resulted in both inconsistent clinical coding and lags in the addition of new clinical codes for the new 6-in-1 vaccine. We excluded these years from subsequent analyses.

A. MMR

Financial year	% children vaccinated								
	Second birthday			Fifth birthday					
	Primary course			Primary course			Full course		
	CPRD	NHS	Difference	CPRD	NHS	Difference	CPRD	NHS	Difference
2008-09	89·3	84·9	4·4						
2009-10	90·8	88·2	2·6						
2010-11	91	89·1	1·9						
2011-12	93·1	91·2	1·9	96·7	92·9	3·8	89·9	86	3·9
2012-13	94·3	92·3	2	97	93·9	3·1	91·3	87·7	3·6
2013-14	95·1	92·7	2·4	97·6	94·1	3·5	92·4	88·3	4·1
2014-15	94·7	92·3	2·4	97·9	94·4	3·5	92·8	88·6	4·2
2015-16	94·3	91·9	2·4	97·9	94·8	3·1	92·5	88·2	4·3
2016-17	94	91·6	2·4	97·8	95	2·8	91·9	87·6	4·3
2017-18	93·1	91·2	1·9	97·5	94·9	2·6	91·5	87·2	4·3
2018-19	92·4	90·3	2·1	97·3	94·5	2·8	90·9	86·4	4·5
2019-20	92·4	90·6	1·8	97·1	94·5	2·6	91	86·8	4·2
2020-21	92·5	90·3	2·2	96·6	94·3	2·3	90·5	86·6	3·9

B. 6/5/4-in1

Financial year	% children vaccinated											
	First birthday			Second birthday			Fifth birthday					
	Primary course (5-in-1 or 6-in-1)									Full course (5-in-1 or 4-in-1)		
	CPRD	NHS	Difference	CPRD	NHS	Difference	CPRD	NHS	Difference	CPRD	NHS	Difference
2007-08	92·9	91·3	1·6									
2008-09	94·4	92	2·4	94·8	93·9	0·9						
2009-10	94·7	93·6	1·1	96·3	95·3	1						
2010-11	94·9	94·2	0·7	96·5	96	0·5						
2011-12	95·4	94·7	0·7	96·8	96·1	0·7	96·1	95·4	0·7	81·3	87·4	-6·1
2012-13	95·9	94·7	1·2	97·1	96·3	0·8	97·4	95·8	1·6	85·8	88·9	-3·1
2013-14	95·9	94·3	1·6	97·3	96·1	1·2	97·6	95·6	2	87·5	88·7	-1·2
2014-15	95·9	94·2	1·7	97·2	95·7	1·5	97·7	95·6	2·1	88·3	88·5	-0·2
2015-16	95·4	93·6	1·8	97·1	95·2	1·9	97·9	95·6	2·3	89·4	86·3	3·1
2016-17	95·3	93·4	1·9	96·7	95·1	1·6	98	95·6	2·4	90	86·2	3·8
2017-18	91	93·1	-2·1	96·4	95·1	1·3	97·8	95·6	2·2	89·8	85·6	4·2
2018-19	59·6	92·1	-32·5	91·4	94·2	-2·8	97·7	95	2·7	89·4	84·8	4·6
2019-20	90·9	92·6	-1·7	60·3	93·8	-33·5	97·1	95·2	1·9	89·3	85·4	3·9
2020-21	91	92	-1	91·9	93·8	-1·9	96·7	95·2	1·5	89	85·3	3·7

C. MenC & Hib/MenC

Financial year	% children vaccinated								
	First birthday			Second birthday			Fifth birthday		
	Primary course (MenC)			Hib/MenC			Hib/MenC		
	CPRD	NHS	Difference	CPRD	NHS	Difference	CPRD	NHS	Difference
2007-08	92·7	90·3	2·4						
2008-09	93·7	91·2	2·5	86	85·4	0·6			
2009-10	93·9	92·7	1·2	91·1	90	1·1			
2010-11	94·4	93·4	1	92·4	91·6	0·8			
2011-12	94·8	93·9	0·9	93·4	92·3	1·1	90·3	88·9	1·4
2012-13	95·3	93·9	1·4	94·2	92·7	1·5	94·3	91·5	2·8
2013-14	83·4			94·5	92·5	2	95·4	91·9	3·5
2014-15	81·8			94·3	92·1	2·2	96·1	92·4	3·7
2015-16	97·1			94·1	91·6	2·5	96·8	92·6	4·2
2016-17	94·2			94	91·5	2·5	96·6	92·6	4
2017-18				93·2	91·2	2	96·6	92·4	4·2
2018-19				92·9	90·4	2·5	96·5	92·2	4·3
2019-20				92·7	90·5	2·2	96·5	92·5	4
2020-21				92·7	90·2	2·5	96·2	92·3	3·9

D. MenB

Financial year	% children vaccinated					
	First birthday			Second birthday		
	Primary course			Full course		
	CPRD	NHS	Difference	CPRD	NHS	Difference
2017-18	94·3	92·5	1·8			
2018-19	93·6	92	1·6	89·8	87·8	2
2019-20	93·5	92·5	1	89	88·7	0·3
2020-21	93·3	92·1	1·2	89·8	89	0·8

E. Rotavirus

Financial year	% children vaccinated		
	First birthday		
	Primary course		
	CPRD	NHS	Difference
2016-17	92·9	89·6	3·3
2017-18	92·7	90·1	2·6
2018-19	92	89·7	2·3
2019-20	91·7	90·1	1·6
2020-21	91·7	90·2	1·5

F. Pneumococcal

Financial year	% children vaccinated					
	First birthday			Second birthday		
	Primary course			Full course		
	CPRD	NHS	Difference	CPRD	NHS	Difference
2007-08	86·1	83·7	2·4			
2008-09	93·6	91·3	2·3	78·5	81·5	-3
2009-10	94·2	92·9	1·3	87·4	87·6	-0·2
2010-11	94·5	93·6	0·9	88·5	89·3	-0·8
2011-12	95·2	94·2	1	91	91·5	-0·5
2012-13	95·7	94·4	1·3	92·6	92·5	0·1
2013-14	95·8	94·1	1·7	93·4	92·4	1
2014-15	95·9	93·9	2	93·1	92·2	0·9
2015-16	95·9	93·5	2·4	93	91·5	1·5
2016-17	95·6	93·5	2·1	92·9	91·5	1·4
2017-18	95·3	93·3	2	92·3	91	1·3
2018-19	94·6	92·8	1·8	91·8	90·2	1·6
2019-20	94·5	93·2	1·3	90·9	90·4	0·5
2020-21				90·7	90·1	0·6

Appendix 4. Algorithm for assigning the most plausible ethnicity & additional methodology for deriving ethnicity

Code lists and data sources

Previously published code lists developed for CPRD GOLD and Aurum (3-5) were combined with additional ethnicity codes from a structured search of the CPRD Aurum code browser version June 2021. Existing GOLD code lists comprising of ReadV2 codes were mapped to SNOMED-CT codes and medcodes using the NHS Digital Data Migration Pack Version April 2020 (6). The code lists and R scripts that we used to identify ethnicity in CPRD Aurum and HES APC have been made available in GitHub.

We used the 'medcode' field of the CPRD Aurum Observations data table and the 'ethnos' field within HES APC episodes to identify ethnic codes. In CPRD Aurum, ethnicity codes are more granular and allow researchers flexibility to disaggregate and aggregate ethnic groups. We grouped iterations of codes that referred to the same ethnic group (e.g. 'Pakistani or British Pakistani - Ethnic Category 2001 Census', 'Pakistani', 'Race: Pakistani') in order to create the 18-level categorisation in the 2011 Census for England and Wales.

In HES APC, ethnicity is more rigidly coded using the 16-level 2001 Census classification system. Additionally, HES ethnicity provided by CPRD as part of standard data linkages is recoded by CPRD into 12 groups. For example, White British, Irish and Any other White background are combined into White (7). As such, we only used HES ethnicity where an individual had no useable ethnic codes in CPRD Aurum. For existing aggregated codes like those in CPRD pre-processed HES data that couldn't be directly assigned a disaggregated category, we categorised them into the most commonly occurring disaggregated 18-level ethnic category within the respective 5-level aggregated category in the general population of England (8), 0-4 years for children and women aged 15-49 for mothers. This gave the greatest likelihood of an individual belonging to the recoded disaggregated category, and reduced likelihood of over-representation of the 'Any other ___ background' category (9).

To align with the end of follow-up, we only used ethnic codes recorded before a child's fifth birthday; for mothers, this was their most recent child's fifth birthday. Ethnic records with no date or implausible dates occurring before the individual's estimated birth date were rare, so we retained and processed them using the algorithm below. Ethnic records with missing or implausible dates could be due to ways that GP practice staff record ethnicity in EMIS. This could hypothetically include situations where parents nominated their child's ethnicity before the child was born, and the date was omitted or filled with a generic 'system missing' date like '1899-12-31'.

Our study started in 2006 to maximise completeness of ethnicity coding as the recording of ethnicity in CPRD markedly improved during this period due to financial incentivisation (5, 10).

Assigning a single most plausible ethnic group

To assign a single most plausible ethnic group for individuals who had multiple ethnicity records (Supplemental figure 2), we adapted the algorithm developed by Mathur (11), and included an additional step from the Public Health England CHIME algorithm: where a patient had discrepant ethnic records that occurred with the same frequency and were recorded either on a) the same date, b) with no date, or c) implausible dates occurring >1 year before the individual's estimated birth date, we assigned their ethnic category that occurred most commonly in the general population of England (again 2011 census, 0-4 years for children and women aged 15-49 years for mothers) (8).

Supplemental figure 2. Algorithm for assigning an individual with a single most plausible ethnicity

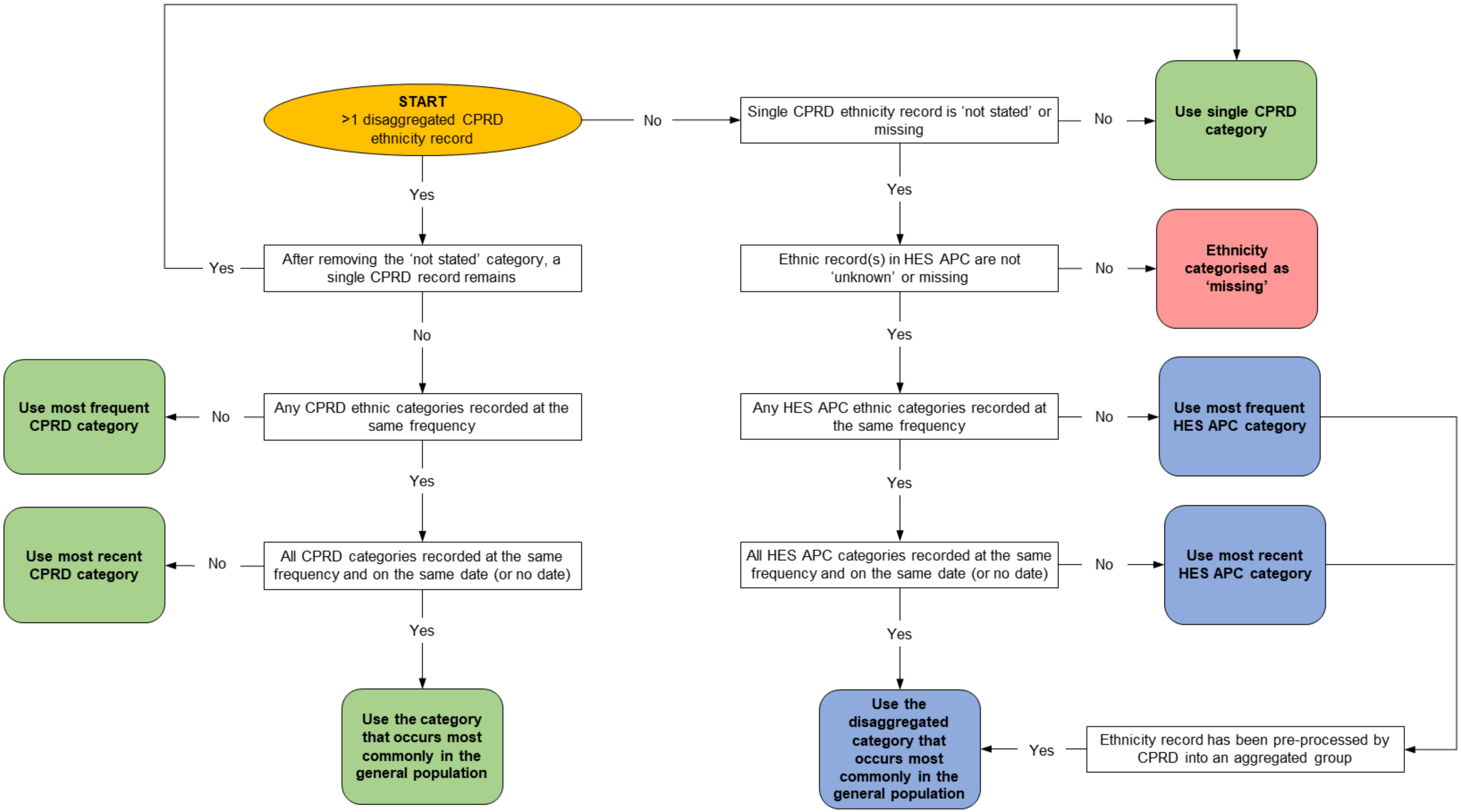

Appendix 5. DAG construction and deriving other study variables

The DAG in Figure 2 primarily draws upon the existing literature (12-20), particularly causal frameworks from 3 recent papers: a US study of ethnicity and influenza vaccination (19), a UK conceptual framework on the impact of COVID-19 on minority ethnic groups (18), and a model of the impact of structural racism on health inequities across the life course with a particular focus on the 'intergenerational transmission' aspects of racism from parents/caregivers to child health outcomes (14). While the explanatory factor was derived using ethnic codes in EHRs, we use the DAG to show that ethnicity recorded in EHRs is determined by (or more likely a proxy for) the multidimensional construct of self-identified ethnicity (18, 21). We also grouped other variables into 'super nodes' (22) for simplicity. We placed racism at two points along the causal path, both proximal to ethnicity, to avoid cyclic feedback loops and to aid visualisation. We acknowledge that this is an oversimplification; in reality, racism both affects and is affected by many of the mediators along the path from ethnicity to a given health outcome.

Aside from the explanatory factor and outcome, we derived the other measured variables in the DAG as per the table below.

**Variable**	**Data source**	**Record**	**Coding**	**Derivation**
Same/different maternal and child ethnicity<br><br>*For cohort description only, not used in modelling*	Aurum, HES APC	Mother, child	Binary: same, different	Comparison of disaggregated categories of maternal and child ethnicity. Not applicable if ethnicity is missing for mother or child.
Sex<br><br>*For cohort description only, not used in modelling*	Aurum	Child	Binary: male, female	Existing variable in Aurum
Year of birth<br><br>*For cohort description only, not used in modelling*	Mother-Baby Link	Child	Categorical: financial year of birth, 2006/07 to 2020/21	Determined by whether the child's estimated delivery date in the Mother-Baby Link was between 1 April and 31 March of a given financial year
Region	Aurum	Child	Categorical:<br>• London<br>• East of England<br>• East Midlands<br>• North East<br>• North West<br>• South East<br>• South West<br>• West Midlands<br>• Yorkshire and The Humber	Existing variable in Aurum using ONS regions
Index of Multiple Deprivation	ONS IMD 2019	Mother, child	Categorical (quintiles): 1 (least deprived), 2, 3, 4, 5 (most deprived)	Existing variable provided by ONS (23), use maternal data if child data is missing
Rural-Urban Classification	ONS RUC 2011	Mother, child	Binary: urban, rural	
Maternal age	Aurum, Mother-Baby Link	Mother, child	Categorical:<br>• Under 20	Difference in years between the child's estimated delivery date in the Mother-Baby Link and mother's estimated date of birth

			• 20-24 years<br>• 25-29 years<br>• 30-34 years<br>• 35-39 years<br>• 40 and older	(set to 30 June of their year of birth as CPRD does not provide date of birth)
Multiple birth<br><br>*Only used to make cohort exclusions, not used in cohort description or modelling*	Mother-Baby Link, Pregnancy Register, HES	Mother, child	Binary: singleton, multiple birth	Any one of:<br>• Children with the same estimated delivery date in Mother-Baby Link, linked to the same mother<br>• Children who were identified as singletons using the Mother-Baby Link but the associated pregnancy (Pregnancy Register pregnancy end date +/- 30 days of Mother-Baby Link estimated delivery date) had multiple non-duplicate records in Pregnancy Register<br>• Children who were identified as singletons using the Mother-Baby Link but the associated delivery in the HES APC maternity dataset (HES APC maternity episode start date +/- 30 days of Mother-Baby Link estimated delivery date) had multiple non-duplicate records even after linking records by baby's sex
Gestational age	Pregnancy Register, HES	Mother, child	Binary: term (37-42 weeks), preterm (23-36 weeks)	The UK-WHO Neonatal and Infant Close Monitoring Growth Chart was used to create gestational age specific plausible upper & lower bounds for birthweight by sex. Implausible values outside of these bounds were removed. Gestational age fields in the Pregnancy Register and the HES APC maternity dataset and birthweight in HES APC maternity dataset were then triangulated, giving preference to values that were concordant between as many data points as possible. Where discrepancies emerged, data from HES was given preference over Pregnancy Register, and gestational age over birthweight.
Birthweight<br><br>*For cohort description only, not used in modelling*	HES	Mother	Categorical:<br>• very low (<1500g)<br>• low (1500-2499g)<br>• normal (2500-3999g)<br>• high (≥4000g)	
Mode of birth	HES	Mother	Binary: spontaneous vaginal, other mode of both (including instrumental, elective caesarean, emergency caesarean and other modes)	Aggregate 10 'delivery method' codes in HES APC maternity dataset into 4 categories
First time mother	Mother-Baby Link, Pregnancy Register	Mother, child	Binary: yes, no	If a child's estimated delivery date in Mother-Baby Link is +/- 30 days of the estimated pregnancy end date of their mother's first live birth in her Pregnancy Register records, this mother is flagged as a first time mother for this child

Appendix 6. Childhood vaccination coverage by ethnicity and financial year of vaccination, and absolute difference comparing each ethnic group with White British

Colour legend for coverage ≤70% ≥95%

A. First birthday

6/5-in-1 primary course<br>% coverage (% difference compared to White British)															
**Maternal ethnicity**	**All years**	**2007-08**	**2008-09**	**2009-10**	**2010-11**	**2011-12**	**2012-13**	**2013-14**	**2014-15**	**2015-16**	**2016-17**	**2017-18**	**2018-19**	**2019-20**	**2020-21**
All	94·2	92·9	94·4	94·8	94·9	95·4	95·9	95·9	95·9	95·4	95·3	91		90·9	91
English, Welsh, Scottish, Northern Irish or British	95	93·3	94·8	95·2	95·1	95·7	96·3	96·4	96·4	96·2	96·2	92·8		92·3	92·8
Irish	93 (2)	93 (0·3)	92·9 (1·9)	93 (2·1)	94·4 (0·7)	92·7 (3)	95·1 (1·1)	94·4 (2)	93·8 (2·6)	95·5 (0·7)	95·2 (1·1)	87·1 (5·7)		89·6 (2·7)	91·3 (1·5)
Any other White background	91·9 (3·1)	92·4 (1)	93·8 (1)	93·1 (2)	94·6 (0·5)	94·9 (0·8)	94·9 (1·4)	94·4 (2·1)	93·9 (2·5)	92·6 (3·6)	92·2 (4)	86·8 (6)		87·1 (5·2)	86·9 (5·9)
Indian	95·5 (-0·5)	94 (-0·7)	94·9 (-0·1)	96·7 (-1·5)	96·5 (-1·4)	96·8 (-1·1)	96·9 (-0·7)	97·2 (-0·8)	97·1 (-0·7)	96·1 (0·1)	96·8 (-0·6)	92·5 (0·3)		92·4 (-0·1)	91·9 (0·9)
Pakistani	93·6 (1·4)	92 (1·3)	94·2 (0·6)	94·4 (0·7)	94·8 (0·3)	94·9 (0·8)	95·3 (1)	95·7 (0·8)	95·4 (1·1)	95 (1·3)	95 (1·2)	87·8 (5)		90·7 (1·6)	90·1 (2·7)
Bangladeshi	93·6 (1·4)	90·4 (2·9)	95·1 (-0·2)	94·5 (0·7)	94·6 (0·5)	94·4 (1·3)	95·3 (1)	95·9 (0·6)	96·6 (-0·1)	95·1 (1·1)	94·6 (1·7)	88·8 (3·9)		91·7 (0·7)	90·4 (2·4)
Chinese	95·3 (-0·3)	92 (1·3)	95·4 (-0·6)	93·7 (1·4)	94·9 (0·2)	96·3 (-0·6)	96·6 (-0·3)	96·3 (0·2)	97·6 (-1·1)	96·3 (-0·1)	96·7 (-0·4)	94·2 (-1·4)		93·9 (-1·6)	92·7 (0·1)
Any other Asian background	95·1 (-0·1)	93·6 (-0·3)	93·8 (1)	95·1 (0)	96·2 (-1·1)	96·6 (-0·9)	96·4 (-0·2)	96 (0·5)	96·5 (-0·1)	96·4 (-0·1)	96·6 (-0·4)	92·2 (0·6)		91·9 (0·4)	92·9 (-0·2)
Caribbean	87·4 (7·6)	88·7 (4·6)	90·1 (4·7)	91·4 (3·7)	92·3 (2·8)	91 (4·7)	91·9 (4·3)	92 (4·5)	89·6 (6·9)	89·7 (6·5)	83 (13·2)	77·3 (15·5)		77·6 (14·8)	70·1 (22·7)
African	93 (2)	92·2 (1·1)	93·3 (1·5)	94·1 (1·1)	94·5 (0·6)	95 (0·7)	95·6 (0·7)	94·6 (1·9)	95·3 (1·2)	94·8 (1·4)	94·6 (1·6)	87·2 (5·6)		86·9 (5·5)	87·4 (5·4)
Any other Black, African or Caribbean background	90·9 (4·1)	91·4 (1·9)	91·2 (3·6)	93·6 (1·6)	92·2 (2·9)	93·9 (1·8)	93·4 (2·8)	93 (3·4)	92·8 (3·7)	93·2 (3)	92 (4·2)	83·7 (9)		82·8 (9·5)	83·9 (8·9)
White and Black Caribbean	89 (6)	88·2 (5·1)	93·1 (1·8)	90·7 (4·4)	90·4 (4·7)	89·6 (6·1)	92·9 (3·4)	91·4 (5·1)	90·5 (5·9)	90·9 (5·4)	88·6 (7·6)	84·4 (8·4)		82·7 (9·7)	82 (10·8)
White and Black African	92·2 (2·8)	88·5 (4·8)	91·7 (3·1)	94·7 (0·4)	93·1 (2)	91·9 (3·8)	93·2 (3·1)	95·6 (0·8)	92·1 (4·4)	93·9 (2·4)	93·8 (2·4)	89·5 (3·3)		90·2 (2·1)	89·2 (3·6)
White and Asian	94·2 (0·8)	95 (-1·7)	93 (1·8)	97·4 (-2·2)	94·7 (0·4)	96·4 (-0·7)	96·3 (0)	92·7 (3·8)	96·7 (-0·3)	92·2 (4)	95·1 (1·2)	89·1 (3·7)		93·9 (-1·5)	93·2 (-0·4)
Any other Mixed or multiple ethnic background	91·3 (3·7)	86·8 (6·5)	91·5 (3·3)	93·2 (2)	91·8 (3·3)	94·4 (1·3)	93·5 (2·7)	94·8 (1·7)	92·9 (3·5)	92·8 (3·5)	91·4 (4·9)	87·5 (5·3)		89·4 (2·9)	86·6 (6·2)
Any other ethnic group	87·8 (7·2)	88·5 (4·8)	89·9 (4·9)	88·8 (6·3)	89·7 (5·4)	90·9 (4·8)	90·4 (5·9)	90·8 (5·6)	91·1 (5·3)	89·8 (6·4)	88·4 (7·9)	82 (10·8)		81·8 (10·5)	81·7 (11·1)
Unknown	92·8 (2·2)	92·2 (1·1)	94·1 (0·7)	93·3 (1·8)	93·9 (1·2)	95·1 (0·7)	95·3 (1)	93·1 (3·4)	93·6 (2·8)	93·9 (2·3)	91·8 (4·4)	89·1 (3·7)		89·3 (3)	90·9 (1·9)

MenC primary course<br>% coverage (% difference compared to White British)															
**Maternal ethnicity**	**All years**	**2007-08**	**2008-09**	**2009-10**	**2010-11**	**2011-12**	**2012-13**	**2013-14**	**2014-15**	**2015-16**	**2016-17**	**2017-18**	**2018-19**	**2019-20**	**2020-21**
All	94·2	92·7	93·7	93·9	94·4	94·8	95·3			97·1	94·2				
English, Welsh, Scottish, Northern Irish or British	93 (1·7)	93·2	94·2	94·4	94·7	95·2	95·8			97·8	95·4				
Irish	93·4 (1·2)	93·6 (-0·4)	92·3 (1·8)	90·6 (3·9)	94·7 (0)	91·3 (3·9)	94·9 (0·9)			97·5 (0·3)	95·4 (0)				
Any other White background	94·8 (-0·2)	92·2 (1)	92·5 (1·7)	92·4 (2)	93·7 (1·1)	94 (1·2)	94·4 (1·4)			94·5 (3·3)	90·9 (4·5)				
Indian	93·3 (1·3)	93·5 (-0·2)	93·4 (0·7)	94·8 (-0·4)	95·1 (-0·4)	95·5 (-0·2)	95·8 (0)			96·9 (0·9)	95·3 (0·1)				
Pakistani	93·9 (0·7)	91·3 (1·9)	92·9 (1·3)	93·2 (1·2)	94·1 (0·7)	94·3 (1)	93·8 (2)			97·2 (0·7)	93·2 (2·1)				
Bangladeshi	93·9 (0·7)	91·1 (2·1)	94·8 (-0·6)	94·1 (0·3)	94·8 (0)	93·4 (1·9)	95·2 (0·6)			97·5 (0·3)	92·8 (2·6)				
Chinese	94·1 (0·5)	90·2 (3)	94·7 (-0·6)	92·5 (1·9)	94·4 (0·3)	94·8 (0·5)	95·6 (0·2)			97·5 (0·4)	95·7 (-0·3)				
Any other Asian background	90·5 (4·1)	92·6 (0·6)	91·9 (2·2)	93·3 (1·2)	94·8 (-0·1)	95·3 (-0·1)	95·7 (0·1)			97·4 (0·4)	95·1 (0·2)				
Caribbean	93·3 (1·3)	89·2 (4)	89·2 (5)	91 (3·5)	91 (3·8)	90·8 (4·5)	91·7 (4·1)			93·1 (4·7)	84·5 (10·9)				
African	91·8 (2·9)	91·1 (2·1)	92·8 (1·4)	92·9 (1·5)	93·6 (1·1)	94·5 (0·8)	94·8 (1)			96·5 (1·3)	92·7 (2·6)				
Any other Black, African or Caribbean background	90·6 (4)	91·2 (2)	90·3 (3·8)	92 (2·4)	91·2 (3·5)	92·7 (2·6)	92·9 (2·9)			96·6 (1·2)	90·9 (4·4)				
White and Black Caribbean	91·5 (3·1)	89·9 (3·4)	92 (2·2)	90·2 (4·2)	89·3 (5·4)	89·5 (5·8)	93·1 (2·7)			95·8 (2)	89·2 (6·2)				
White and Black African	94·1 (0·5)	87·6 (5·6)	89·8 (4·4)	92·5 (1·9)	92·5 (2·2)	91·9 (3·3)	93·2 (2·6)			95·9 (1·9)	91·8 (3·5)				
White and Asian	91·6 (3)	92·5 (0·7)	90·9 (3·3)	96 (-1·6)	94·2 (0·5)	95 (0·3)	94·8 (1)			95·1 (2·8)	93·3 (2·1)				
Any other Mixed or multiple ethnic background	88·8 (5·8)	87·4 (5·9)	91·2 (2·9)	92·5 (2)	90·6 (4·1)	93·8 (1·4)	92·3 (3·5)			94·5 (3·3)	91·4 (4)				
Any other ethnic group	92·9 (1·7)	86·5 (6·8)	88·6 (5·6)	88·1 (6·3)	88·6 (6·1)	90·3 (5)	89·9 (5·9)			92·6 (5·2)	86·5 (8·8)				
Unknown	93 (1·7)	92·4 (0·8)	93·1 (1·1)	92·2 (2·3)	93·3 (1·4)	93·5 (1·7)	95 (0·8)			95·4 (2·4)	91·5 (3·9)				

MenB primary course<br>% coverage (% difference compared to White British)															
**Maternal ethnicity**	**All years**	**2007-08**	**2008-09**	**2009-10**	**2010-11**	**2011-12**	**2012-13**	**2013-14**	**2014-15**	**2015-16**	**2016-17**	**2017-18**	**2018-19**	**2019-20**	**2020-21**
All	93·8										94·3	94·3	93·6	93·5	93·3
English, Welsh, Scottish, Northern Irish or British	95·4										95·6	95·9	95·4	95·1	95·2
Irish	93·2 (2·2)										93·1 (2·5)	92·8 (3·1)	92·4 (3)	92·9 (2·1)	94·9
Any other White background	89·6 (5·9)										90·4 (5·1)	90 (5·9)	89·1 (6·3)	89·5 (5·5)	94·8 (0·4)
Indian	95 (0·4)										94·9 (0·6)	95 (0·9)	94·8 (0·6)	95·4 (-0·3)	89·1 (6·1)
Pakistani	93 (2·4)										93·9 (1·7)	92·6 (3·3)	92·9 (2·5)	93·8 (1·3)	95 (0·2)
Bangladeshi	93·3 (2·2)										93·9 (1·7)	93·2 (2·7)	93·2 (2·2)	94·2 (0·8)	92·3 (2·9)
Chinese	95·9 (-0·4)										94·2 (1·4)	95·7 (0·2)	97 (-1·6)	96·3 (-1·3)	92 (3·2)
Any other Asian background	94·8 (0·7)										95·9 (-0·3)	94·4 (1·5)	95·4 (0)	94·1 (1)	95·5 (-0·3)
Caribbean	77·2 (18·2)										80·7 (14·9)	81 (14·9)	74·9 (20·5)	78·4 (16·7)	94·5 (0·7)
African	90·8 (4·7)										92·9 (2·6)	91·2 (4·7)	90·8 (4·6)	89·9 (5·2)	72·1 (23·1)
Any other Black, African or Caribbean background	87·8 (7·6)										90·8 (4·7)	90·2 (5·7)	87·5 (7·9)	85·5 (9·6)	89·7 (5·5)
White and Black Caribbean	86·4 (9)										88·3 (7·2)	88·3 (7·6)	86·7 (8·7)	85·3 (9·8)	85·6 (9·6)
White and Black African	91·1 (4·3)										95·6 (0)	91·8 (4·1)	85·9 (9·5)	93 (2·1)	84·2 (11)
White and Asian	93·6 (1·8)										95 (0·6)	92·4 (3·5)	93·2 (2·2)	94·2 (0·9)	91 (4·3)
Any other Mixed or multiple ethnic background	89·6 (5·9)										88·8 (6·7)	91 (4·9)	87·7 (7·7)	91·5 (3·6)	94·2 (1·1)
Any other ethnic group	84·7 (10·8)										85·6 (10)	85·7 (10·2)	83·7 (11·7)	85 (10·1)	88·8 (6·4)
Unknown	91·4 (4)										90·6 (5)	91·6 (4·3)	90·3 (5·1)	91·1 (3·9)	83·8 (11·4)

Rotavirus primary course<br>% coverage (% difference compared to White British)															
**Maternal ethnicity**	**All years**	**2007-08**	**2008-09**	**2009-10**	**2010-11**	**2011-12**	**2012-13**	**2013-14**	**2014-15**	**2015-16**	**2016-17**	**2017-18**	**2018-19**	**2019-20**	**2020-21**
All	92·1								91·2	92·4	92·9	92·7	92·0	91·7	91·7
English, Welsh, Scottish, Northern Irish or British	93·8								92·3	93·6	94·4	94·5	94·1	93·5	93·9
Irish	90·7 (3·1)								83·9 (8·4)	92·3 (1·3)	91·4 (3)	90·2 (4·3)	92·4 (1·7)	90·9 (2·7)	92·3 (1·6)
Any other White background	88·1 (5·7)								89·3 (2·9)	89·3 (4·3)	88·7 (5·7)	88·1 (6·3)	87·2 (6·9)	87·4 (6·1)	87·1 (6·7)
Indian	94·1 (-0·3)								93 (-0·8)	93·7 (-0·2)	94·8 (-0·4)	94·8 (-0·3)	93·9 (0·2)	94·5 (-0·9)	93·6 (0·2)
Pakistani	89·3 (4·5)								88·3 (3·9)	90·2 (3·4)	90·8 (3·6)	90·3 (4·2)	87·6 (6·4)	89·8 (3·7)	87·9 (6)
Bangladeshi	89·9 (3·9)								90·5 (1·8)	88·6 (4·9)	91·1 (3·3)	90·9 (3·6)	90·3 (3·8)	89·5 (4)	88·2 (5·6)
Chinese	94·9 (-1·1)								93·6 (-1·3)	94·8 (-1·2)	94·5 (-0·1)	95·7 (-1·2)	95·9 (-1·8)	94·9 (-1·3)	94·5 (-0·7)
Any other Asian background	92·9 (0·9)								91·5 (0·8)	92·7 (0·8)	93·5 (0·9)	92·6 (1·9)	94·1 (0)	93 (0·5)	92·6 (1·2)
Caribbean	78·6 (15·2)								81·6 (10·7)	84·1 (9·5)	81·9 (12·5)	79·5 (15)	74·5 (19·6)	77·3 (16·2)	71·5 (22·4)
African	89·4 (4·3)								89·2 (3·1)	91 (2·6)	91·3 (3·1)	90·1 (4·4)	88·6 (5·5)	87·3 (6·2)	88·2 (5·7)
Any other Black, African or Caribbean background	85·7 (8·1)								84·9 (7·4)	88·9 (4·7)	88·6 (5·8)	86·2 (8·3)	84·2 (9·9)	82·6 (10·9)	83·4 (10·4)
White and Black Caribbean	85·2 (8·6)								83·7 (8·5)	89·6 (4)	86·8 (7·6)	86·7 (7·8)	85 (9·1)	83·1 (10·4)	81·6 (12·3)
White and Black African	88·8 (5)								86·6 (5·7)	91·5 (2·1)	90·5 (3·9)	90·8 (3·7)	84·6 (9·5)	88·1 (5·4)	88·9 (5)
White and Asian	91·3 (2·5)								89·8 (2·5)	89·4 (4·2)	93·3 (1·1)	90·7 (3·8)	90·4 (3·7)	93·2 (0·3)	91·9 (2)
Any other Mixed or multiple ethnic background	87·8 (6)								87·4 (4·9)	87·2 (6·4)	88·5 (5·9)	89·9 (4·6)	86·2 (7·9)	88·7 (4·8)	87 (6·8)
Any other ethnic group	81·8 (12)								83·7 (8·6)	83·4 (10·2)	83·1 (11·3)	82 (12·5)	80 (14·1)	81·6 (11·9)	79·6 (14·3)
Unknown	89·9 (3·9)								88·7 (3·5)	91 (2·5)	89·2 (5·2)	89·9 (4·6)	89·8 (4·3)	88·9 (4·7)	91·3 (2·5)

Pneumococcal primary course<br>% coverage (% difference compared to White British)															
**Maternal ethnicity**	**All years**	**2007-08**	**2008-09**	**2009-10**	**2010-11**	**2011-12**	**2012-13**	**2013-14**	**2014-15**	**2015-16**	**2016-17**	**2017-18**	**2018-19**	**2019-20**	**2020-21**
All	94·4	86·1	93·7	94·2	94·5	95·2	95·7	95·8	95·9	95·9	95·6	95·3	94·6	94·5	94·2
English, Welsh, Scottish, Northern Irish or British	95·1	86·8	94·1	94·7	94·8	95·5	96·1	96·4	96·5	96·7	96·6	96·7	96·2	95·9	95·9
Irish	93·5 (1·6)	87 (-0·2)	92·3 (1·8)	91·9 (2·8)	94·9 (-0·2)	92·1 (3·4)	94·7 (1·4)	94·4 (1·9)	94·3 (2·3)	95·3 (1·4)	95·6 (1)	94·3 (2·4)	92·9 (3·3)	93·1 (2·7)	94·6 (1·3)
Any other White background	92·4 (2·7)	86 (0·9)	92·7 (1·4)	92·7 (2)	94 (0·8)	94·7 (0·8)	94·7 (1·5)	94·3 (2)	93·9 (2·6)	93 (3·7)	92·3 (4·3)	91·3 (5·4)	90·5 (5·6)	90·7 (5·2)	90·1 (5·7)
Indian	95·6 (-0·5)	86·1 (0·8)	92·8 (1·4)	95·3 (-0·6)	96·1 (-1·3)	95·7 (-0·2)	96·6 (-0·5)	97·1 (-0·7)	97·1 (-0·6)	96·5 (0·2)	97·3 (-0·7)	96·9 (-0·2)	96·7 (-0·6)	97·1 (-1·3)	96·2 (-0·4)
Pakistani	93·8 (1·3)	83·4 (3·4)	93·3 (0·8)	93·9 (0·8)	94·2 (0·6)	94·4 (1·1)	95 (1·1)	95·2 (1·2)	95·4 (1·1)	95·6 (1·1)	95·6 (1)	94·3 (2·4)	93·8 (2·4)	94·6 (1·2)	93·2 (2·7)
Bangladeshi	93·9 (1·2)	83·4 (3·4)	94 (0·1)	94·2 (0·5)	94·4 (0·4)	94·6 (0·9)	95·1 (1·1)	95·7 (0·7)	96·7 (-0·1)	95·5 (1·2)	95·3 (1·3)	94·6 (2·1)	94·2 (2)	94·6 (1·3)	92·2 (3·6)
Chinese	95·4 (-0·3)	86·1 (0·7)	93·8 (0·3)	92·2 (2·5)	94·6 (0·2)	95·2 (0·3)	96·8 (-0·6)	95·5 (0·8)	97·2 (-0·7)	96·5 (0·2)	96·5 (0·1)	96·6 (0·1)	98·7 (-2·5)	97·6 (-1·7)	95·8 (0)
Any other Asian background	95·1 (0)	86·2 (0·6)	93·3 (0·8)	94 (0·7)	95·4 (-0·6)	96 (-0·4)	96·1 (0)	95·9 (0·5)	96·2 (0·3)	96·7 (0)	96·7 (0)	96·2 (0·4)	96·6 (-0·4)	95·5 (0·3)	95·3 (0·5)
Caribbean	86·4 (8·7)	78 (8·8)	90·2 (3·9)	90·9 (3·8)	91 (3·8)	91 (4·5)	91·6 (4·5)	92·6 (3·7)	89·7 (6·8)	90·4 (6·3)	82·8 (13·8)	82·1 (14·6)	75·6 (20·5)	80·4 (15·5)	74·4 (21·5)
African	92·9 (2·2)	83 (3·8)	92·2 (1·9)	93 (1·7)	93·9 (0·9)	94·7 (0·9)	95·4 (0·7)	94·2 (2·2)	95·3 (1·2)	95·4 (1·3)	95·2 (1·5)	93·1 (3·5)	92·5 (3·6)	90·9 (5)	90·8 (5·1)
Any other Black, African or Caribbean background	90·8 (4·3)	80·6 (6·2)	90·1 (4)	92·7 (2)	92·2 (2·6)	92·7 (2·8)	93·8 (2·3)	93·4 (3)	93·2 (3·4)	93·8 (2·9)	92·8 (3·8)	90·7 (6)	88·3 (7·8)	87·3 (8·6)	88·3 (7·6)
White and Black Caribbean	88·8 (6·3)	79·5 (7·3)	91·3 (2·8)	89·2 (5·5)	89·7 (5·1)	90·2 (5·3)	92·4 (3·7)	91 (5·4)	90·5 (6)	91·7 (5)	89 (7·6)	89·5 (7·1)	88·7 (7·5)	85·7 (10·2)	85·3 (10·5)
White and Black African	92·1 (3)	79·8 (7)	90·4 (3·7)	93·8 (0·9)	92·5 (2·3)	91·9 (3·6)	92·5 (3·6)	94·2 (2·1)	90·8 (5·7)	94·9 (1·8)	93·8 (2·8)	93·4 (3·3)	89·6 (6·6)	93·9 (2)	93·1 (2·8)
White and Asian	94·3 (0·8)	82·5 (4·3)	90·9 (3·3)	96 (-1·3)	94·7 (0·1)	94·6 (0·9)	96·3 (-0·2)	93·7 (2·7)	96·7 (-0·2)	92·9 (3·8)	96·1 (0·5)	94 (2·6)	94·1 (2·1)	96·5 (-0·6)	95·5 (0·4)
Any other Mixed or multiple ethnic background	91·5 (3·6)	78·7 (8·2)	90 (4·1)	91·8 (2·9)	91·2 (3·6)	94·7 (0·8)	93·5 (2·6)	94·8 (1·6)	92·9 (3·6)	93 (3·7)	91·9 (4·7)	93·4 (3·3)	89·3 (6·8)	92·2 (3·7)	89·8 (6·1)
Any other ethnic group	88·3 (6·8)	81·7 (5·1)	88·7 (5·4)	88·4 (6·3)	89·1 (5·7)	90·8 (4·7)	90·7 (5·4)	91·1 (5·3)	91·2 (5·4)	89·7 (6·9)	88·8 (7·9)	87·3 (9·4)	85·3 (10·8)	86·5 (9·3)	85·7 (10·2)
Unknown	91·9 (3·3)	85·8 (1)	93·7 (0·4)	93·4 (1·3)	93·5 (1·3)	95·2 (0·3)	96 (0·1)	93·2 (3·2)	93·3 (3·2)	93·6 (3·1)	92·8 (3·8)	92·6 (4·1)	91·9 (4·3)	91·9 (4)	93·5 (2·3)

B. Second birthday

MMR primary course<br>% coverage (% difference compared to White British)															
**Maternal ethnicity**	**All years**	**2007-08**	**2008-09**	**2009-10**	**2010-11**	**2011-12**	**2012-13**	**2013-14**	**2014-15**	**2015-16**	**2016-17**	**2017-18**	**2018-19**	**2019-20**	**2020-21**
All	92·9		89·3	90·8	91·0	93·1	94·3	95·1	94·7	94·3	94·0	93·1	92·4	92·4	92·5
English, Welsh, Scottish, Northern Irish or British	94·3		90·1	91·7	91·9	93·8	95·2	96·1	95·8	95·7	95·8	95·2	95·0	95·2	95·3
Irish	89·2 (5·1)		85·4 (4·7)	88·2 (3·5)	88·1 (3·8)	89·1 (4·7)	88·3 (6·9)	93·5 (2·7)	91·9 (3·8)	93·8 (1·8)	89·9 (5·8)	89·3 (5·9)	84·5 (10·4)	87·1 (8·1)	88·6 (6·7)
Any other White background	88·8 (5·5)		87·4 (2·7)	88 (3·7)	89·3 (2·5)	91·3 (2·4)	92·9 (2·4)	91·9 (4·3)	91·7 (4·1)	90·8 (4·9)	89 (6·8)	87·1 (8·1)	85·3 (9·6)	85 (10·2)	85·4 (9·9)
Indian	94·8 (-0·5)		93 (-2·9)	92·7 (-0·9)	94·1 (-2·3)	95·7 (-1·9)	94·7 (0·5)	95·9 (0·2)	95·2 (0·6)	95·9 (-0·3)	94·1 (1·6)	94·8 (0·4)	95 (0)	95·4 (-0·2)	95·8 (-0·4)
Pakistani	93·3 (1)		90·6 (-0·5)	91·5 (0·2)	92·4 (-0·6)	94·6 (-0·9)	94·8 (0·4)	95·1 (1)	95·2 (0·6)	93·4 (2·3)	93·5 (2·2)	93 (2·2)	93·1 (1·8)	92·3 (2·9)	92·8 (2·6)
Bangladeshi	93·4 (0·8)		90·9 (-0·9)	92·7 (-1)	91·3 (0·5)	94·8 (-1)	94·1 (1·1)	95·5 (0·6)	96·4 (-0·7)	94·4 (1·2)	94·5 (1·2)	92·3 (2·9)	91·8 (3·1)	92·8 (2·4)	92·6 (2·8)
Chinese	92·7 (1·5)		87·1 (3)	90·1 (1·6)	90·4 (1·4)	90·8 (3)	92 (3·3)	93 (3·1)	94·2 (1·6)	92·9 (2·8)	92·8 (2·9)	94·6 (0·6)	94 (1)	96·4 (-1·2)	95·5 (-0·2)
Any other Asian background	93·5 (0·7)		90·7 (-0·6)	91·2 (0·5)	92·2 (-0·3)	94·7 (-1)	94·5 (0·7)	94·7 (1·4)	94·2 (1·5)	94·4 (1·2)	93·7 (2)	92·7 (2·5)	94·3 (0·7)	93·8 (1·4)	93·7 (1·7)
Caribbean	81·3 (13)		78·5 (11·6)	82·6 (9·1)	81·1 (10·7)	86·6 (7·1)	87·5 (7·7)	89·6 (6·6)	87·2 (8·5)	85·8 (9·8)	82·2 (13·5)	76·3 (18·9)	72·5 (22·4)	68·9 (26·3)	70 (25·3)
African	86 (8·2)		83·7 (6·4)	84·7 (7·1)	85·2 (6·6)	87·9 (5·9)	88·8 (6·4)	89·9 (6·3)	88·8 (7)	88·2 (7·5)	88·8 (7)	85·4 (9·8)	81·7 (13·2)	82·3 (12·9)	80·5 (14·8)
Any other Black, African or Caribbean background	76·7 (17·6)		76·6 (13·4)	78 (13·7)	75·3 (16·5)	78·4 (15·4)	82·2 (13)	82·3 (13·8)	80·5 (15·3)	80·9 (14·7)	79·7 (16)	75·2 (20)	69 (26)	65·7 (29·5)	67·5 (27·8)
White and Black Caribbean	85·8 (8·5)		80·7 (9·4)	85 (6·7)	84·1 (7·7)	86·5 (7·3)	89·5 (5·7)	89·9 (6·2)	92·4 (3·4)	87·3 (8·4)	85·9 (9·9)	85·2 (10)	85·4 (9·6)	83·1 (12·1)	79·8 (15·5)
White and Black African	88 (6·2)		81·3 (8·8)	85·3 (6·4)	83·2 (8·6)	88·2 (5·6)	89 (6·2)	92·6 (3·5)	93·1 (2·7)	88·4 (7·3)	92·4 (3·3)	89·6 (5·6)	87·1 (7·8)	83·7 (11·5)	87·2 (8·1)
White and Asian	93·5 (0·7)		90·5 (-0·4)	89·5 (2·2)	93·1 (-1·3)	96·8 (-3)	95·3 (-0·1)	94 (2·2)	95·5 (0·3)	96·7 (-1·1)	93·3 (2·5)	91·9 (3·3)	92·4 (2·6)	92·7 (2·5)	92·9 (2·4)
Any other Mixed or multiple ethnic background	88·5 (5·7)		83 (7·1)	84·7 (7)	84·8 (7)	90·2 (3·5)	89·8 (5·4)	91·7 (4·4)	91·5 (4·3)	92·7 (2·9)	89·8 (6)	86·8 (8·4)	88 (7)	86·2 (9)	88·6 (6·7)
Any other ethnic group	87·1 (7·2)		86 (4·1)	85·8 (5·9)	84·3 (7·6)	88·3 (5·5)	89·2 (6)	90·5 (5·7)	90·3 (5·5)	88·8 (6·9)	87·7 (8)	85 (10·2)	85·7 (9·2)	86·5 (8·7)	83·8 (11·5)
Unknown	90·3 (4)		86·9 (3·2)	90·8 (0·9)	89·5 (2·3)	90·1 (3·7)	94·3 (1)	94·6 (1·6)	92·8 (2·9)	92·6 (3)	92·9 (2·8)	91·3 (3·9)	90·1 (4·9)	90·2 (5)	89·6 (5·8)

**Hib/MenC<br>% coverage (% difference compared to White British)**															
**Maternal ethnicity**	**All years**	**2007-08**	**2008-09**	**2009-10**	**2010-11**	**2011-12**	**2012-13**	**2013-14**	**2014-15**	**2015-16**	**2016-17**	**2017-18**	**2018-19**	**2019-20**	**2020-21**
All	92·7		86	91·1	92·4	93·4	94·2	94·5	94·3	94·1	94	93·2	92·9	92·7	92·7
English, Welsh, Scottish, Northern Irish or British	94·1		87	92	93·1	94·1	95·2	95·6	95·3	95·3	95·6	95·2	95·3	95·3	95·4
Irish	89·6 (4·5)		82·1 (4·9)	87·6 (4·4)	92·7 (0·5)	90·8 (3·3)	89·4 (5·8)	93·2 (2·3)	91·9 (3·3)	93·5 (1·7)	88·8 (6·8)	90·2 (5)	86·3 (8·9)	87·9 (7·5)	88·6 (6·8)
Any other White background	88·7 (5·4)		83 (4)	88·3 (3·7)	89·7 (3·5)	91·3 (2·8)	92·2 (3)	91·4 (4·2)	91·4 (3·9)	90·7 (4·5)	89 (6·6)	87·3 (7·9)	86 (9·2)	85·6 (9·8)	85·7 (9·7)
Indian	93·8 (0·3)		87·5 (-0·5)	91·9 (0·1)	93·5 (-0·4)	94·3 (-0·2)	93·8 (1·4)	94·8 (0·8)	94·4 (0·9)	94·8 (0·5)	93·8 (1·8)	94·3 (0·9)	94·7 (0·6)	95·7 (-0·3)	96 (-0·6)
Pakistani	93·2 (0·9)		86·8 (0·3)	91·5 (0·5)	94 (-0·9)	94·2 (-0·1)	94·8 (0·4)	94·8 (0·7)	94·5 (0·8)	94 (1·3)	93·2 (2·3)	93·1 (2·1)	93·7 (1·5)	92·5 (2·8)	92·9 (2·6)
Bangladeshi	93·3 (0·8)		86·8 (0·3)	92·7 (-0·7)	91·9 (1·2)	95·8 (-1·7)	93·4 (1·8)	95·5 (0)	96·2 (-1)	94·4 (0·8)	94·8 (0·8)	92·8 (2·4)	92·3 (3)	93·1 (2·2)	92·8 (2·7)
Chinese	90·5 (3·6)		79·5 (7·6)	86·9 (5·1)	88·1 (5)	87 (7·1)	89·4 (5·8)	89·8 (5·8)	91·7 (3·5)	90·4 (4·8)	91·7 (3·9)	93·3 (1·9)	93·5 (1·8)	96·2 (-0·8)	95·3 (0·1)
Any other Asian background	92·9 (1·2)		87·2 (-0·2)	90·1 (1·9)	91·7 (1·4)	94·2 (-0·1)	93·9 (1·3)	93·9 (1·6)	93·1 (2·1)	93·9 (1·4)	93·7 (1·9)	92·1 (3·1)	94·7 (0·5)	94·3 (1)	94 (1·5)
Caribbean	82·1 (12)		77·1 (9·9)	84·8 (7·2)	85·3 (7·9)	87·8 (6·3)	89·1 (6)	89·7 (5·9)	87·5 (7·8)	85·2 (10)	82·8 (12·7)	76·4 (18·8)	71·6 (23·6)	69·3 (26)	71·4 (24)
African	87·4 (6·7)		80 (7)	87·6 (4·4)	89 (4·1)	90·5 (3·6)	90 (5·1)	90·3 (5·3)	89·6 (5·7)	89·8 (5·5)	89·8 (5·8)	86·9 (8·3)	83·9 (11·4)	83·8 (11·6)	81·9 (13·5)
Any other Black, African or Caribbean background	81·9 (12·2)		79·7 (7·3)	81·3 (10·7)	84·3 (8·9)	87·2 (6·9)	86·2 (8·9)	86·7 (8·9)	85 (10·2)	84·3 (11)	83·8 (11·8)	81·9 (13·3)	76·9 (18·4)	70·8 (24·5)	70·6 (24·8)
White and Black Caribbean	86·7 (7·4)		79 (8·1)	88·3 (3·7)	87·9 (5·2)	88·4 (5·7)	91·1 (4·1)	89·7 (5·8)	92·4 (2·9)	87·3 (8)	87 (8·5)	86·2 (9)	85·8 (9·4)	83·3 (12·1)	80·2 (15·2)
White and Black African	89·6 (4·5)		81·3 (5·7)	90 (2)	89·1 (4·1)	91·1 (3)	89·9 (5·2)	91·9 (3·7)	94·7 (0·6)	90·1 (5·1)	91·6 (3·9)	91·1 (4·1)	88·3 (7)	85·3 (10)	87·2 (8·2)
White and Asian	93·2 (0·9)		85 (2)	90·1 (1·9)	94·6 (-1·4)	95·2 (-1·1)	94·1 (1·1)	93·6 (2)	95·1 (0·2)	96·7 (-1·5)	93·7 (1·9)	91·5 (3·7)	92·4 (2·9)	93·1 (2·3)	93·3 (2·2)
Any other Mixed or multiple ethnic background	88·6 (5·5)		78·6 (8·5)	86·3 (5·7)	88·9 (4·3)	90·9 (3·2)	90 (5·2)	91·3 (4·2)	93·1 (2·2)	91·5 (3·8)	89·3 (6·2)	86 (9·2)	87·3 (7·9)	85·7 (9·7)	88·6 (6·8)
Any other ethnic group	86·1 (8)		81·3 (5·7)	86·1 (5·9)	85·9 (7·3)	88·2 (5·9)	88·5 (6·7)	89 (6·6)	89·1 (6·1)	88·4 (6·9)	87·3 (8·3)	83·9 (11·3)	84·6 (10·7)	84·2 (11·2)	83·1 (12·3)
Unknown	88·4 (5·7)		80·9 (6·1)	87·9 (4·1)	89·4 (3·7)	89·4 (4·7)	94·7 (0·5)	93·6 (2)	92·1 (3·1)	91·5 (3·8)	92·9 (2·6)	91·9 (3·3)	90·2 (5)	90·7 (4·6)	89·2 (6·2)

MenB full course<br>% coverage (% difference compared to White British)															
**Maternal ethnicity**	**All years**	**2007-08**	**2008-09**	**2009-10**	**2010-11**	**2011-12**	**2012-13**	**2013-14**	**2014-15**	**2015-16**	**2016-17**	**2017-18**	**2018-19**	**2019-20**	**2020-21**
All	89·6											90·4	89·8	89·0	89·8
English, Welsh, Scottish, Northern Irish or British	92·3											93·0	92·5	91·7	92·5
Irish	84·7 (7·7)											83·1 (9·9)	82·7 (9·7)	85·9 (5·8)	86·2 (6·3)
Any other White background	82·7 (9·7)											83·4 (9·6)	82·9 (9·6)	81·9 (9·7)	82·8 (9·7)
Indian	91·2 (1·1)											90·4 (2·6)	90·7 (1·8)	91 (0·6)	92·5 (0)
Pakistani	89·7 (2·7)											88·2 (4·8)	89·8 (2·7)	89·8 (1·9)	90·4 (2·1)
Bangladeshi	89·9 (2·4)											90·4 (2·6)	89 (3·5)	90 (1·7)	90·6 (1·9)
Chinese	91·6 (0·8)											89·6 (3·4)	89·8 (2·7)	93·8 (-2·1)	92·4 (0)
Any other Asian background	91 (1·4)											89·4 (3·6)	91·2 (1·3)	91·4 (0·3)	91·2 (1·3)
Caribbean	67·3 (25)											72·2 (20·8)	67·6 (24·8)	64·1 (27·6)	67·6 (24·9)
African	80·1 (12·2)											82·8 (10·2)	79·3 (13·1)	79·6 (12·1)	79·8 (12·7)
Any other Black, African or Caribbean background	70·4 (22)											76·2 (16·8)	72·4 (20·1)	67·6 (24·1)	67·5 (25)
White and Black Caribbean	80·1 (12·2)											82·1 (10·9)	82·2 (10·3)	79·7 (12)	77·4 (15·1)
White and Black African	83·6 (8·8)											87·7 (5·3)	84·5 (8)	78·6 (13·1)	84·8 (7·6)
White and Asian	89·5 (2·9)											89·6 (3·4)	89·1 (3·4)	89·6 (2·1)	89·7 (2·8)
Any other Mixed or multiple ethnic background	84·3 (8)											84·9 (8·1)	83·9 (8·6)	81·8 (9·9)	87·1 (5·4)
Any other ethnic group	78·4 (14)											78·8 (14·2)	79·2 (13·3)	77·1 (14·5)	78·5 (13·9)
Unknown	87·2 (5·2)											87·2 (5·8)	88 (4·5)	86·9 (4·7)	86·6 (5·9)

Pneumococcal full course
% coverage (% difference compared to White British)

Maternal ethnicity	All years	2007-08	2008-09	2009-10	2010-11	2011-12	2012-13	2013-14	2014-15	2015-16	2016-17	2017-18	2018-19	2019-20	2020-21
All	90·5		78·5	87·4	88·5	91·0	92·6	93·4	93·1	93·0	92·9	92·3	91·8	90·9	90·7
English, Welsh, Scottish, Northern Irish or British	91·9		79·9	88·7	89·6	91·8	93·5	94·5	94·2	94·3	94·6	94·4	94·4	93·6	93·4
Irish	87·3 (4·6)		73·8 (6)	82 (6·7)	85·6 (3·9)	88·8 (3)	88·5 (5)	91 (3·5)	90·2 (4)	92·6 (1·7)	88·8 (5·8)	89·7 (4·7)	85·6 (8·8)	86·4 (7·2)	86·7 (6·7)
Any other White background	86·9 (5)		75·2 (4·7)	84·4 (4·3)	86·4 (3·1)	89·2 (2·6)	91·2 (2·4)	90·5 (4)	90·5 (3·7)	89·3 (5)	88 (6·6)	86·1 (8·2)	84·8 (9·6)	84 (9·6)	83·8 (9·6)
Indian	92·2 (-0·3)		78·8 (1·1)	87·7 (0·9)	91·1 (-1·6)	93·6 (-1·8)	92·2 (1·4)	94·1 (0·4)	94·1 (0·2)	94·7 (-0·4)	93 (1·6)	94·1 (0·3)	94·4 (-0·1)	94·3 (-0·7)	94·6 (-1·2)
Pakistani	90·1 (1·8)		75·9 (4)	86·9 (1·7)	88·7 (0·8)	91·3 (0·4)	92 (1·5)	92·9 (1·5)	92·4 (1·8)	92·4 (1·8)	92·3 (2·3)	91·6 (2·8)	92 (2·4)	90·9 (2·7)	91·1 (2·3)
Bangladeshi	90·7 (1·2)		76·2 (3·7)	88·5 (0·2)	88·5 (1)	92·7 (-0·9)	91·7 (1·8)	93·4 (1·1)	94·9 (-0·7)	93·2 (1·1)	93·2 (1·4)	92·1 (2·3)	91·3 (3·1)	91·4 (2·1)	90·6 (2·8)
Chinese	89·9 (2)		75·2 (4·6)	84·8 (3·8)	86 (3·6)	88·1 (3·7)	89·7 (3·8)	90·7 (3·7)	90·6 (3·6)	92·3 (1·9)	91·2 (3·4)	93·3 (1·1)	91·7 (2·7)	95·8 (-2·2)	94·1 (-0·7)
Any other Asian background	91·3 (0·6)		79·6 (0·3)	87·9 (0·8)	89 (0·6)	92·4 (-0·7)	92·7 (0·8)	93·1 (1·4)	92·2 (2)	93 (1·2)	92·8 (1·8)	91·6 (2·8)	93·9 (0·4)	93·3 (0·3)	92·8 (0·6)
Caribbean	77·5 (14·4)		65·1 (14·8)	76·8 (11·9)	77·7 (11·8)	83·6 (8·2)	86·6 (6·9)	86·1 (8·4)	85·7 (8·5)	83·6 (10·7)	80·3 (14·3)	73·4 (21)	69·2 (25·2)	65·2 (28·4)	68·4 (25)
African	84·2 (7·7)		70 (9·9)	79·7 (9)	82·4 (7·2)	86·4 (5·3)	88·8 (4·8)	89·1 (5·3)	88·2 (6·1)	88·5 (5·8)	88·9 (5·7)	86 (8·4)	82·3 (12·1)	82·1 (11·5)	80·4 (13)
Any other Black, African or Caribbean background	76·9 (15)		64·9 (15)	73 (15·6)	73·4 (16·2)	78·9 (12·8)	83·6 (9·9)	84·3 (10·1)	81·8 (12·4)	82·2 (12·1)	82·5 (12·1)	80·7 (13·7)	74·2 (20·2)	68·9 (24·7)	67·8 (25·6)
White and Black Caribbean	81·8 (10·1)		67 (12·8)	79·7 (9)	78·7 (10·9)	82·9 (8·8)	86·5 (7·1)	86·5 (7·9)	88·8 (5·5)	85·7 (8·5)	83·3 (11·3)	83·3 (11·1)	84·2 (10·1)	80·9 (12·6)	77·2 (16·2)
White and Black African	86 (5·9)		67·4 (12·5)	82·6 (6)	81·8 (7·8)	87·9 (3·9)	87·4 (6·1)	90 (4·5)	92·5 (1·7)	85·6 (8·7)	89·7 (4·9)	90·7 (3·7)	86·4 (8)	82·5 (11)	85·9 (7·5)
White and Asian	91·1 (0·8)		74·1 (5·7)	84·2 (4·4)	91·1 (-1·6)	94·1 (-2·3)	92·9 (0·6)	92·4 (2·1)	92·8 (1·4)	95·1 (-0·8)	91·7 (2·9)	91·9 (2·5)	92 (2·4)	92 (1·5)	91·5 (1·9)
Any other Mixed or multiple ethnic background	85·8 (6·1)		67·3 (12·5)	79·2 (9·4)	82 (7·5)	87·6 (4·2)	89·6 (3·9)	89·3 (5·1)	90·7 (3·6)	90 (4·3)	87·4 (7·2)	86 (8·4)	86·5 (7·8)	83·7 (9·8)	86·9 (6·4)
Any other ethnic group	82·8 (9·1)		72·8 (7·1)	80·9 (7·8)	81·5 (8·1)	84·7 (7·1)	85·9 (7·6)	86·4 (8·1)	87 (7·2)	86·1 (8·2)	85·2 (9·4)	82·9 (11·5)	81·3 (13·1)	79·6 (13·9)	79·6 (13·7)
Unknown	86·7 (5·2)		78 (1·9)	86·7 (1·9)	88·6 (1)	88·1 (3·7)	92·4 (1·2)	93·2 (1·3)	90·7 (3·5)	91·1 (3·1)	91·8 (2·8)	90 (4·4)	89 (5·4)	88·2 (5·4)	86·8 (6·6)

C. Fifth birthday

MMR full course<br>% coverage (% difference compared to White British)															
**Maternal ethnicity**	**All years**	**2007-08**	**2008-09**	**2009-10**	**2010-11**	**2011-12**	**2012-13**	**2013-14**	**2014-15**	**2015-16**	**2016-17**	**2017-18**	**2018-19**	**2019-20**	**2020-21**
All	91·5					89·9	91·3	92·4	92·8	92·5	92·0	91·5	90·9	91·0	90·5
English, Welsh, Scottish, Northern Irish or British	93·2					90·9	92·2	93·4	93·8	93·9	93·7	93·5	93·3	93·8	93·4
Irish	87·1 (6·1)					87·9 (2·9)	89·2 (3)	91·3 (2·1)	88·4 (5·3)	87·7 (6·2)	87·5 (6·1)	84·8 (8·7)	86·5 (6·7)	83 (10·8)	85·9 (7·5)
Any other White background	84·2 (8·9)					85·3 (5·5)	87·3 (4·9)	87·6 (5·8)	88·6 (5·2)	86·6 (7·3)	85·1 (8·5)	84·1 (9·4)	81·9 (11·3)	80·1 (13·7)	79·6 (13·8)
Indian	92·1 (1)					92·3 (-1·4)	91·7 (0·5)	93·6 (-0·3)	93·8 (-0·1)	92·1 (1·8)	91·6 (2)	90·8 (2·7)	91·8 (1·4)	90·8 (3)	92·8 (0·6)
Pakistani	91·6 (1·6)					91 (-0·1)	91·9 (0·3)	92·3 (1·1)	93·1 (0·6)	93 (0·9)	90·6 (3·1)	91·2 (2·3)	90·1 (3·1)	91·3 (2·5)	91 (2·4)
Bangladeshi	91·1 (2·1)					91·7 (-0·8)	92·6 (-0·5)	92·9 (0·5)	92·6 (1·1)	92·2 (1·7)	90·7 (2·9)	92 (1·5)	89 (4·3)	89·7 (4·1)	87 (6·4)
Chinese	91·1 (2)					90·1 (0·7)	90·6 (1·5)	87·9 (5·5)	91·6 (2·2)	91·8 (2·1)	92·4 (1·2)	92·1 (1·3)	89 (4·2)	90·8 (3·1)	93·7 (-0·3)
Any other Asian background	90·6 (2·6)					90·2 (0·7)	89·5 (2·6)	91·9 (1·5)	92·2 (1·6)	90·9 (3)	91·2 (2·5)	89·8 (3·7)	89·7 (3·6)	89·2 (4·6)	90·7 (2·8)
Caribbean	79·3 (13·8)					78·6 (12·3)	84·1 (8·1)	83·3 (10)	84·9 (8·9)	86·6 (7·3)	81·5 (12·2)	77·5 (16)	72·5 (20·7)	72·6 (21·3)	64·8 (28·7)
African	84·9 (8·2)					82·1 (8·7)	86·4 (5·8)	88·4 (4·9)	89·3 (4·5)	87·4 (6·5)	85·7 (7·9)	84·5 (9)	83·3 (10)	81·9 (12)	79·1 (14·3)
Any other Black, African or Caribbean background	78·1 (15·1)					76·1 (14·8)	83·5 (8·7)	82·8 (10·6)	81·6 (12·2)	79·7 (14·2)	80·4 (13·3)	77·2 (16·3)	74·1 (19·1)	73 (20·8)	71·6 (21·9)
White and Black Caribbean	83·8 (9·4)					80 (10·8)	88·2 (4)	88·4 (5)	88·5 (5·2)	88 (5·9)	85·7 (8)	83·1 (10·4)	79 (14·3)	77·7 (16·1)	78·1 (15·3)
White and Black African	85·6 (7·5)					84·8 (6)	85·1 (7·1)	88·4 (5)	85·8 (8)	87·4 (6·5)	84 (9·6)	85·7 (7·8)	82·6 (10·6)	84·8 (9)	87·1 (6·4)
White and Asian	91·4 (1·8)					88·3 (2·6)	85·6 (6·6)	96·2 (-2·8)	94 (-0·2)	94·1 (-0·2)	90·3 (3·3)	91·3 (2·1)	92·1 (1·1)	88·7 (5·2)	91·2 (2·2)
Any other Mixed or multiple ethnic background	86·4 (6·8)					83·1 (7·8)	88·2 (3·9)	88·3 (5·1)	90·9 (2·9)	85·6 (8·3)	84·3 (9·4)	87·1 (6·4)	83·1 (10·2)	87·7 (6·1)	85·2 (8·2)
Any other ethnic group	83·2 (9·9)					83·7 (7·2)	80·9 (11·3)	86·4 (6·9)	88·6 (5·2)	84·5 (9·4)	84·3 (9·4)	81·2 (12·3)	82·3 (11)	82·5 (11·4)	79·4 (14·1)
Unknown	89·3 (3·9)					86·7 (4·2)	90·3 (1·9)	91·4 (1·9)	90 (3·8)	92·4 (1·5)	90·1 (3·6)	89·3 (4·2)	88·8 (4·5)	89·9 (4)	87·8 (5·6)

5/4-in-1 full course<br>% coverage (% difference compared to White British)															
**Maternal ethnicity**	**All years**	**2007-08**	**2008-09**	**2009-10**	**2010-11**	**2011-12**	**2012-13**	**2013-14**	**2014-15**	**2015-16**	**2016-17**	**2017-18**	**2018-19**	**2019-20**	**2020-21**
All	88					81·3	85·8	87·5	88·3	89·4	90	89·8	89·4	89·3	89
English, Welsh, Scottish, Northern Irish or British	89·9					82·7	87	88·7	89·3	91	92	92	92·1	92·5	92·2
Irish	83·7 (6·2)					79·7 (2·9)	82·7 (4·3)	86·7 (2)	84·3 (5)	86·2 (4·8)	86·1 (5·9)	81·6 (10·5)	81·8 (10·3)	83 (9·4)	84 (8·2)
Any other White background	80·9 (9)					74·6 (8·1)	81·7 (5·4)	83 (5·8)	84·6 (4·7)	83·7 (7·3)	82·7 (9·2)	81·7 (10·3)	80 (12·1)	77·6 (14·9)	77·6 (14·6)
Indian	87·3 (2·6)					79·1 (3·6)	82·8 (4·3)	86·6 (2·1)	88 (1·4)	88·4 (2·6)	88·5 (3·5)	87·9 (4·1)	89·8 (2·3)	89·2 (3·2)	91·5 (0·7)
Pakistani	86·5 (3·4)					79 (3·7)	83·4 (3·7)	84·1 (4·6)	86·3 (3·1)	88·4 (2·6)	88·2 (3·8)	88·2 (3·8)	88·1 (4)	89·7 (2·8)	89 (3·2)
Bangladeshi	86·9 (3)					80 (2·7)	84·6 (2·4)	86·4 (2·4)	88 (1·3)	88·3 (2·6)	88·6 (3·4)	89·7 (2·3)	86·8 (5·3)	88·8 (3·6)	87 (5·2)
Chinese	87·2 (2·6)					79·9 (2·7)	83·9 (3·1)	83·8 (4·9)	86·6 (2·7)	88·7 (2·3)	87·3 (4·7)	89·3 (2·8)	88·3 (3·8)	89·5 (3)	90·9 (1·3)
Any other Asian background	86·7 (3·2)					80·1 (2·6)	84·1 (2·9)	85·4 (3·3)	86·7 (2·7)	88 (3)	88·7 (3·3)	88 (4)	88 (4·1)	87·3 (5·2)	88·5 (3·7)
Caribbean	74·2 (15·7)					71·8 (10·9)	79 (8·1)	78·4 (10·3)	79·4 (9·9)	80·8 (10·2)	76·9 (15)	73·7 (18·3)	67·2 (24·9)	66·7 (25·7)	61·3 (30·9)
African	80·9 (8·9)					72·7 (9·9)	80·3 (6·7)	83·4 (5·4)	85·5 (3·8)	83·6 (7·4)	84·1 (7·9)	82·8 (9·3)	79·8 (12·3)	78·1 (14·4)	77·1 (15·1)
Any other Black, African or Caribbean background	76·1 (13·8)					70·7 (11·9)	78·9 (8·1)	78·8 (9·9)	76·8 (12·5)	79·2 (11·8)	80·4 (11·6)	78·4 (13·6)	71·3 (20·8)	72·5 (20)	72·2 (20·1)
White and Black Caribbean	79·9 (10)					72·2 (10·5)	83·7 (3·4)	84·1 (4·6)	82·8 (6·5)	84·6 (6·4)	82·7 (9·3)	80·1 (12)	76·9 (15·2)	74·2 (18·3)	76·8 (15·5)
White and Black African	82·4 (7·5)					77·3 (5·4)	79 (8)	85·9 (2·9)	82·4 (7)	84·3 (6·6)	83 (9)	83·1 (8·9)	80·3 (11·8)	81·3 (11·1)	85·1 (7·1)
White and Asian	87·5 (2·3)					80·2 (2·5)	80 (7)	91·1 (-2·4)	90·6 (-1·3)	90·1 (0·9)	88·8 (3·2)	86·5 (5·5)	91·1 (1)	84·2 (8·2)	88·3 (3·9)
Any other Mixed or multiple ethnic background	82·4 (7·5)					70·5 (12·1)	79·6 (7·4)	84·4 (4·3)	85·5 (3·9)	83·3 (7·6)	82·7 (9·3)	84·3 (7·7)	80·8 (11·3)	85·8 (6·6)	81·6 (10·6)
Any other ethnic group	79·2 (10·7)					76·1 (6·6)	76·2 (10·8)	82·2 (6·5)	83·5 (5·9)	80·1 (10·9)	80·8 (11·2)	78·3 (13·7)	79·7 (12·4)	79·3 (13·1)	75·4 (16·8)
Unknown	85·2 (4·6)					80·4 (2·2)	85·1 (1·9)	87·6 (1·1)	86·6 (2·8)	88·9 (2)	87 (4·9)	87·9 (4·1)	86·6 (5·5)	88·1 (4·4)	86·9 (5·3)

Appendix 7. Main analysis: Average (total) effect of maternal ethnicity on MMR childhood vaccination, accounting for effect modification by time period where statistically significant (comparing each ethnic group with the White British reference group in the same time period)

A. MMR

**Maternal ethnicity**	**RR (95%CI)**				
	2008-09 to 2010-2011	2011-12 to 2013-14	2014-15 to 2016-17	2017-18 to 2019-20	2020-21
	**Primary course (second birthday)**				
Irish	0·96 (0·93-0·98)	0·95 (0·9-1)	0·96 (0·91-1·01)	0·92 (0·86-0·97)	0·93 (0·87-0·99)
Any other White background	0·97 (0·96-0·97)	0·97 (0·95-0·98)	0·95 (0·93-0·96)	0·9 (0·89-0·92)	0·9 (0·88-0·91)
Indian	1·02 (1·02-1·03)	1 (0·99-1·02)	0·99 (0·98-1·01)	1 (0·98-1·01)	1 (0·99-1·02)
Pakistani	1 (1-1·01)	1 (0·98-1·01)	0·98 (0·97-1)	0·98 (0·96-0·99)	0·97 (0·95-0·99)
Bangladeshi	1·01 (0·99-1·02)	1 (0·97-1·02)	0·99 (0·97-1·02)	0·97 (0·95-1)	0·97 (0·94-1)
Chinese	0·98 (0·96-1)	0·97 (0·93-1·01)	0·97 (0·94-1·01)	1 (0·96-1·04)	1 (0·96-1·05)
Any other Asian background	1 (0·99-1·01)	1 (0·98-1·01)	0·98 (0·96-1)	0·98 (0·96-1)	0·98 (0·96-1·01)
Caribbean	0·88 (0·87-0·9)	0·92 (0·89-0·96)	0·89 (0·85-0·93)	0·76 (0·72-0·8)	0·73 (0·68-0·79)
African	0·93 (0·92-0·93)	0·93 (0·92-0·95)	0·93 (0·91-0·94)	0·87 (0·86-0·89)	0·84 (0·82-0·87)
Any other Black, African or Caribbean background	0·84 (0·82-0·86)	0·85 (0·8-0·91)	0·84 (0·79-0·9)	0·73 (0·68-0·79)	0·71 (0·64-0·78)
White and Black Caribbean	0·91 (0·89-0·93)	0·93 (0·89-0·98)	0·93 (0·88-0·98)	0·89 (0·84-0·94)	0·84 (0·78-0·9)
White and Black African	0·92 (0·89-0·95)	0·95 (0·88-1·02)	0·95 (0·89-1·02)	0·91 (0·85-0·98)	0·92 (0·84-1)
White and Asian	1 (0·97-1·03)	1 (0·94-1·06)	0·99 (0·94-1·05)	0·97 (0·91-1·03)	0·97 (0·91-1·04)
Any other Mixed or multiple ethnic background	0·92 (0·9-0·95)	0·95 (0·9-1·01)	0·95 (0·9-1·01)	0·91 (0·86-0·97)	0·93 (0·87-0·99)
Any other ethnic group	0·94 (0·92-0·95)	0·94 (0·91-0·97)	0·93 (0·9-0·96)	0·9 (0·87-0·93)	0·88 (0·85-0·91)
Unknown	0·97 (0·96-0·98)	0·97 (0·95-1)	0·97 (0·94-1)	0·95 (0·92-0·98)	0·94 (0·9-0·98)
	**Full course (fifth birthday)**				
Irish		0·97 (0·95-1)	0·94 (0·88-0·99)	0·91 (0·85-0·97)	0·92 (0·85-0·99)
Any other White background		0·94 (0·94-0·95)	0·92 (0·91-0·94)	0·88 (0·86-0·89)	0·85 (0·83-0·87)
Indian		1 (1-1·01)	0·99 (0·97-1)	0·97 (0·96-0·99)	0·99 (0·97-1·02)
Pakistani		1 (0·99-1)	0·98 (0·97-1)	0·97 (0·95-0·99)	0·97 (0·95-1)
Bangladeshi		1 (0·99-1·01)	0·98 (0·95-1·01)	0·96 (0·94-0·99)	0·93 (0·89-0·97)
Chinese		0·97 (0·95-0·99)	0·98 (0·93-1·03)	0·97 (0·92-1·02)	1 (0·95-1·06)
Any other Asian background		0·98 (0·97-0·99)	0·97 (0·95-1)	0·96 (0·93-0·98)	0·97 (0·94-1)
Caribbean		0·89 (0·87-0·91)	0·9 (0·86-0·94)	0·79 (0·75-0·84)	0·69 (0·63-0·76)
African		0·93 (0·92-0·94)	0·93 (0·91-0·96)	0·89 (0·87-0·91)	0·85 (0·82-0·88)
Any other Black, African or Caribbean background		0·88 (0·85-0·9)	0·86 (0·8-0·92)	0·8 (0·74-0·86)	0·77 (0·69-0·85)
White and Black Caribbean		0·93 (0·9-0·95)	0·93 (0·88-0·99)	0·86 (0·8-0·91)	0·84 (0·77-0·91)
White and Black African		0·94 (0·9-0·97)	0·92 (0·84-0·99)	0·9 (0·83-0·98)	0·93 (0·84-1·03)
White and Asian		0·98 (0·95-1·01)	0·99 (0·92-1·06)	0·97 (0·9-1·04)	0·98 (0·9-1·06)
Any other Mixed or multiple ethnic background		0·94 (0·92-0·97)	0·93 (0·87-0·99)	0·92 (0·86-0·98)	0·91 (0·84-0·99)
Any other ethnic group		0·91 (0·89-0·93)	0·91 (0·88-0·95)	0·88 (0·84-0·91)	0·85 (0·81-0·89)
Unknown		0·96 (0·95-0·98)	0·97 (0·94-1)	0·95 (0·92-0·99)	0·94 (0·9-0·99)

B. 6/5/4-in-1

Maternal ethnicity	RR (95%CI)					
	2007-08	2008-09 to 2010-2011	2011-12 to 2013-14	2014-15 to 2016-17	2017-18 to 2019-20	2020-21
	**Primary course (first birthday)**					
Irish	1 (0·97-1·03)	0·98 (0·92-1·05)	0·98 (0·92-1·04)	0·98 (0·93-1·05)	0·96 (0·89-1·02)	0·98 (0·92-1·06)
Any other White background	0·99 (0·98-1)	0·99 (0·97-1·01)	0·99 (0·97-1)	0·96 (0·95-0·98)	0·94 (0·92-0·96)	0·94 (0·92-0·96)
Indian	1·01 (1-1·02)	1·01 (0·99-1·03)	1·01 (0·99-1·03)	1 (0·98-1·03)	1 (0·98-1·02)	0·99 (0·96-1·02)
Pakistani	0·99 (0·97-1)	0·99 (0·97-1·02)	0·99 (0·97-1·02)	0·99 (0·96-1·01)	0·96 (0·94-0·99)	0·97 (0·94-1)
Bangladeshi	0·97 (0·95-0·99)	1 (0·96-1·04)	0·99 (0·95-1·03)	0·99 (0·95-1·03)	0·97 (0·93-1·02)	0·97 (0·93-1·02)
Chinese	0·99 (0·96-1·01)	1 (0·94-1·05)	1 (0·95-1·06)	1·01 (0·95-1·06)	1·02 (0·96-1·08)	1 (0·94-1·06)
Any other Asian background	1 (0·99-1·02)	1 (0·98-1·03)	1 (0·98-1·03)	1 (0·98-1·03)	0·99 (0·97-1·02)	1 (0·97-1·03)
Caribbean	0·95 (0·93-0·97)	0·96 (0·92-1·01)	0·95 (0·91-1)	0·91 (0·87-0·96)	0·84 (0·79-0·89)	0·76 (0·7-0·82)
African	0·99 (0·98-1)	0·99 (0·97-1·01)	0·99 (0·97-1·01)	0·99 (0·97-1·01)	0·94 (0·92-0·96)	0·94 (0·92-0·97)
Any other Black, African or Caribbean background	0·98 (0·96-1)	0·97 (0·92-1·02)	0·97 (0·92-1·02)	0·96 (0·91-1·02)	0·9 (0·85-0·96)	0·9 (0·84-0·97)
White and Black Caribbean	0·95 (0·92-0·97)	0·96 (0·9-1·02)	0·95 (0·89-1·01)	0·93 (0·88-0·99)	0·9 (0·84-0·97)	0·88 (0·82-0·95)
White and Black African	0·95 (0·9-1)	0·98 (0·89-1·08)	0·97 (0·88-1·07)	0·97 (0·88-1·07)	0·97 (0·88-1·08)	0·96 (0·86-1·07)
White and Asian	1·02 (0·98-1·05)	1 (0·93-1·08)	0·99 (0·92-1·07)	0·98 (0·91-1·06)	0·99 (0·91-1·07)	1 (0·92-1·09)
Any other Mixed or multiple ethnic background	0·93 (0·89-0·97)	0·97 (0·89-1·05)	0·98 (0·9-1·06)	0·96 (0·88-1·04)	0·96 (0·88-1·04)	0·93 (0·85-1·02)
Any other ethnic group	0·95 (0·93-0·97)	0·94 (0·9-0·98)	0·94 (0·91-0·98)	0·93 (0·89-0·97)	0·88 (0·85-0·93)	0·88 (0·84-0·92)
Unknown	0·99 (0·98-1)	0·99 (0·96-1·01)	0·98 (0·96-1·01)	0·97 (0·94-0·99)	0·96 (0·93-1)	0·98 (0·94-1·02)
	**Full course (fifth birthday)**					
Irish			0·96 (0·93-1)	0·94 (0·87-1·02)	0·89 (0·82-0·96)	0·91 (0·83-1)
Any other White background			0·93 (0·92-0·94)	0·92 (0·9-0·94)	0·87 (0·85-0·89)	0·84 (0·82-0·87)
Indian			0·96 (0·95-0·97)	0·97 (0·95-1)	0·97 (0·94-0·99)	0·99 (0·96-1·02)
Pakistani			0·96 (0·94-0·97)	0·97 (0·94-0·99)	0·96 (0·94-0·99)	0·96 (0·93-1)
Bangladeshi			0·97 (0·96-0·99)	0·97 (0·94-1·01)	0·96 (0·92-1)	0·94 (0·9-0·99)
Chinese			0·96 (0·93-0·99)	0·96 (0·9-1·03)	0·97 (0·91-1·03)	0·99 (0·92-1·06)
Any other Asian background			0·97 (0·95-0·98)	0·97 (0·94-1)	0·95 (0·92-0·98)	0·96 (0·92-1)
Caribbean			0·89 (0·87-0·91)	0·87 (0·82-0·92)	0·75 (0·71-0·8)	0·66 (0·6-0·74)
African			0·92 (0·9-0·93)	0·93 (0·9-0·96)	0·87 (0·84-0·9)	0·84 (0·8-0·87)
Any other Black, African or Caribbean background			0·88 (0·85-0·91)	0·87 (0·8-0·94)	0·81 (0·74-0·87)	0·78 (0·7-0·87)
White and Black Caribbean			0·93 (0·9-0·95)	0·92 (0·86-0·98)	0·84 (0·78-0·9)	0·83 (0·76-0·91)
White and Black African			0·94 (0·9-0·98)	0·92 (0·83-1·01)	0·89 (0·8-0·98)	0·92 (0·82-1·03)
White and Asian			0·98 (0·94-1·02)	0·99 (0·9-1·09)	0·95 (0·86-1·04)	0·96 (0·86-1·07)
Any other Mixed or multiple ethnic background			0·92 (0·88-0·95)	0·92 (0·85-1)	0·91 (0·84-0·99)	0·89 (0·8-0·98)
Any other ethnic group			0·91 (0·89-0·93)	0·9 (0·86-0·94)	0·86 (0·82-0·9)	0·82 (0·77-0·87)
Unknown			0·97 (0·96-0·98)	0·96 (0·93-1)	0·95 (0·91-0·99)	0·94 (0·89-0·99)

C. MenC and Hib/MenC

Maternal ethnicity	RR (95%CI)				
	2008-09 to 2010-2011	2011-12 to 2013-14	2014-15 to 2016-17	2017-18 to 2019-20	2020-21
	**Primary course MenC (first birthday)**				
Irish	0·99 (0·98-1)				
Any other White background	0·98 (0·98-0·98)				
Indian	1 (1-1)				
Pakistani	0·99 (0·98-0·99)				
Bangladeshi	0·99 (0·99-1)				
Chinese	1 (0·99-1)				
Any other Asian background	1 (0·99-1)				
Caribbean	0·95 (0·94-0·96)				
African	0·98 (0·98-0·99)				
Any other Black, African or Caribbean background	0·97 (0·96-0·98)				
White and Black Caribbean	0·96 (0·95-0·97)				
White and Black African	0·97 (0·96-0·98)				
White and Asian	0·99 (0·98-1)				
Any other Mixed or multiple ethnic background	0·97 (0·96-0·98)				
Any other ethnic group	0·94 (0·93-0·94)				
Unknown	0·98 (0·97-0·98)				
	**Hib/MenC (second birthday)**				
Irish	0·97 (0·94-0·99)	0·96 (0·91-1·01)	0·96 (0·91-1·01)	0·93 (0·88-0·98)	0·93 (0·87-0·99)
Any other White background	0·96 (0·96-0·97)	0·97 (0·95-0·98)	0·95 (0·93-0·96)	0·91 (0·89-0·92)	0·9 (0·88-0·91)
Indian	1 (1-1·01)	0·99 (0·98-1·01)	0·99 (0·97-1·01)	1 (0·98-1·01)	1·01 (0·99-1·03)
Pakistani	1 (0·99-1·01)	1 (0·98-1·01)	0·98 (0·97-1)	0·98 (0·96-0·99)	0·97 (0·95-0·99)
Bangladeshi	1 (0·99-1·01)	1 (0·97-1·03)	1 (0·97-1·02)	0·97 (0·95-1)	0·97 (0·94-1)
Chinese	0·94 (0·92-0·96)	0·94 (0·89-0·98)	0·96 (0·91-1)	0·99 (0·94-1·04)	1 (0·95-1·05)
Any other Asian background	0·99 (0·98-1)	0·99 (0·97-1·01)	0·98 (0·96-1)	0·98 (0·96-1)	0·98 (0·96-1·01)
Caribbean	0·91 (0·89-0·92)	0·94 (0·9-0·97)	0·89 (0·86-0·93)	0·76 (0·72-0·8)	0·75 (0·7-0·8)
African	0·94 (0·94-0·95)	0·95 (0·93-0·97)	0·94 (0·92-0·96)	0·89 (0·87-0·91)	0·86 (0·83-0·88)
Any other Black, African or Caribbean background	0·9 (0·88-0·92)	0·91 (0·86-0·96)	0·88 (0·84-0·94)	0·8 (0·75-0·85)	0·74 (0·67-0·81)
White and Black Caribbean	0·94 (0·92-0·96)	0·95 (0·9-0·99)	0·93 (0·89-0·98)	0·89 (0·85-0·94)	0·84 (0·78-0·9)
White and Black African	0·96 (0·94-0·99)	0·96 (0·9-1·02)	0·97 (0·91-1·03)	0·93 (0·87-0·99)	0·91 (0·84-0·99)
White and Asian	1 (0·97-1·02)	0·99 (0·93-1·06)	1 (0·94-1·06)	0·97 (0·91-1·03)	0·98 (0·91-1·05)
Any other Mixed or multiple ethnic background	0·94 (0·91-0·96)	0·96 (0·9-1·01)	0·96 (0·91-1·01)	0·91 (0·86-0·96)	0·93 (0·87-0·99)
Any other ethnic group	0·93 (0·92-0·94)	0·93 (0·9-0·96)	0·93 (0·9-0·96)	0·88 (0·86-0·91)	0·87 (0·84-0·91)
Unknown	0·94 (0·93-0·95)	0·97 (0·94-1)	0·97 (0·94-1)	0·95 (0·93-0·98)	0·93 (0·9-0·98)

D. MenB

Maternal ethnicity	RR (95%CI)
	**Primary course (first birthday)**
Irish	0·98 (0·97-0·99)
Any other White background	0·94 (0·94-0·94)
Indian	1 (0·99-1)
Pakistani	0·97 (0·97-0·98)
Bangladeshi	0·98 (0·97-0·98)
Chinese	1 (1-1·01)
Any other Asian background	0·99 (0·99-1)
Caribbean	0·81 (0·79-0·82)
African	0·95 (0·95-0·96)
Any other Black, African or Caribbean background	0·92 (0·91-0·93)
White and Black Caribbean	0·91 (0·89-0·92)
White and Black African	0·95 (0·94-0·97)
White and Asian	0·98 (0·97-0·99)
Any other Mixed or multiple ethnic background	0·94 (0·93-0·95)
Any other ethnic group	0·89 (0·88-0·9)
Unknown	0·96 (0·95-0·97)
	**Full course (second birthday)**
Irish	0·92 (0·9-0·94)
Any other White background	0·91 (0·91-0·92)
Indian	1 (0·99-1·01)
Pakistani	0·99 (0·98-1)
Bangladeshi	1·02 (1·01-1·03)
Chinese	1 (0·99-1·01)
Any other Asian background	1·01 (1-1·02)
Caribbean	0·77 (0·75-0·79)
African	0·91 (0·9-0·92)
Any other Black, African or Caribbean background	0·8 (0·77-0·82)
White and Black Caribbean	0·89 (0·87-0·92)
White and Black African	0·93 (0·9-0·95)
White and Asian	0·98 (0·96-1)
Any other Mixed or multiple ethnic background	0·94 (0·92-0·95)
Any other ethnic group	0·87 (0·86-0·89)
Unknown	0·95 (0·93-0·96)

E. Rotavirus

**Maternal ethnicity**	**RR (95%CI)**
	**Primary course (first birthday)**
Irish	0·97 (0·96-0·98)
Any other White background	0·94 (0·94-0·94)
Indian	1 (1-1·01)
Pakistani	0·95 (0·95-0·96)
Bangladeshi	0·96 (0·95-0·97)
Chinese	1·01 (1-1·02)
Any other Asian background	0·99 (0·99-1)
Caribbean	0·84 (0·83-0·85)
African	0·95 (0·95-0·96)
Any other Black, African or Caribbean background	0·91 (0·9-0·93)
White and Black Caribbean	0·91 (0·9-0·92)
White and Black African	0·95 (0·93-0·96)
White and Asian	0·97 (0·96-0·99)
Any other Mixed or multiple ethnic background	0·94 (0·93-0·95)
Any other ethnic group	0·87 (0·86-0·88)
Unknown	0·96 (0·95-0·97)

F. Pneumococcal

Maternal ethnicity	RR (95%CI)					
	2007-08	2008-09 to 2010-2011	2011-12 to 2013-14	2014-15 to 2016-17	2017-18 to 2019-20	2020-21
	**Primary course (first birthday)**					
Irish	1 (0·96-1·04)	0·99 (0·9-1·07)	0·98 (0·9-1·06)	0·98 (0·9-1·07)	0·97 (0·89-1·06)	0·99 (0·9-1·08)
Any other White background	0·99 (0·98-1)	0·99 (0·96-1·01)	0·99 (0·96-1·01)	0·96 (0·94-0·99)	0·94 (0·92-0·97)	0·94 (0·91-0·97)
Indian	0·99 (0·98-1·01)	1 (0·97-1·04)	1·01 (0·97-1·04)	1 (0·97-1·04)	1·01 (0·97-1·04)	1 (0·97-1·04)
Pakistani	0·96 (0·94-0·98)	0·99 (0·96-1·03)	0·99 (0·95-1·03)	0·99 (0·95-1·03)	0·98 (0·94-1·02)	0·97 (0·93-1·01)
Bangladeshi	0·96 (0·94-0·99)	1 (0·94-1·05)	0·99 (0·94-1·05)	0·99 (0·94-1·05)	0·98 (0·93-1·04)	0·96 (0·91-1·02)
Chinese	0·99 (0·96-1·03)	0·99 (0·92-1·07)	1 (0·93-1·07)	1 (0·93-1·08)	1·01 (0·94-1·09)	1 (0·93-1·08)
Any other Asian background	0·99 (0·97-1·01)	1 (0·96-1·04)	1 (0·96-1·04)	1 (0·96-1·04)	1 (0·96-1·04)	0·99 (0·95-1·04)
Caribbean	0·9 (0·87-0·93)	0·96 (0·9-1·03)	0·96 (0·89-1·02)	0·91 (0·85-0·97)	0·82 (0·77-0·89)	0·78 (0·71-0·85)
African	0·96 (0·94-0·97)	0·98 (0·95-1·02)	0·99 (0·96-1·02)	0·99 (0·96-1·02)	0·96 (0·93-0·99)	0·95 (0·91-0·98)
Any other Black, African or Caribbean background	0·93 (0·89-0·97)	0·97 (0·89-1·05)	0·97 (0·9-1·05)	0·97 (0·89-1·05)	0·92 (0·85-1)	0·92 (0·84-1·01)
White and Black Caribbean	0·92 (0·88-0·95)	0·95 (0·88-1·03)	0·95 (0·87-1·03)	0·94 (0·86-1·02)	0·91 (0·84-0·99)	0·89 (0·81-0·98)
White and Black African	0·92 (0·86-0·98)	0·98 (0·85-1·12)	0·97 (0·85-1·11)	0·96 (0·84-1·1)	0·96 (0·84-1·1)	0·97 (0·84-1·12)
White and Asian	0·95 (0·88-1·02)	0·99 (0·86-1·15)	0·99 (0·85-1·14)	0·99 (0·85-1·14)	0·99 (0·85-1·14)	1 (0·86-1·15)
Any other Mixed or multiple ethnic background	0·91 (0·86-0·96)	0·96 (0·86-1·08)	0·98 (0·88-1·1)	0·96 (0·86-1·07)	0·95 (0·85-1·06)	0·94 (0·83-1·05)
Any other ethnic group	0·94 (0·92-0·97)	0·94 (0·89-0·99)	0·95 (0·9-1)	0·93 (0·88-0·98)	0·9 (0·85-0·95)	0·89 (0·84-0·95)
Unknown	0·99 (0·97-1)	0·99 (0·96-1·02)	0·99 (0·95-1·02)	0·96 (0·93-1)	0·96 (0·92-0·99)	0·98 (0·94-1·02)
	**Full course (second birthday)**					
Irish		0·94 (0·91-0·97)	0·96 (0·9-1·03)	0·96 (0·9-1·03)	0·93 (0·87-0·99)	0·93 (0·86-1·01)
Any other White background		0·96 (0·95-0·97)	0·97 (0·95-0·99)	0·95 (0·93-0·96)	0·9 (0·89-0·92)	0·9 (0·88-0·92)
Indian		1 (0·99-1·01)	1 (0·98-1·02)	1 (0·98-1·02)	1 (0·98-1·02)	1·01 (0·99-1·04)
Pakistani		0·98 (0·97-0·99)	0·99 (0·97-1·01)	0·98 (0·96-1)	0·97 (0·95-0·99)	0·98 (0·95-1)
Bangladeshi		0·98 (0·97-1)	0·99 (0·96-1·03)	0·99 (0·96-1·03)	0·97 (0·94-1·01)	0·97 (0·93-1·01)
Chinese		0·96 (0·94-0·98)	0·96 (0·91-1·01)	0·97 (0·92-1·02)	0·99 (0·94-1·05)	1·01 (0·95-1·07)
Any other Asian background		1 (0·99-1·01)	0·99 (0·97-1·02)	0·98 (0·96-1·01)	0·99 (0·96-1·01)	0·99 (0·97-1·02)
Caribbean		0·85 (0·83-0·87)	0·92 (0·87-0·96)	0·88 (0·84-0·93)	0·73 (0·69-0·78)	0·73 (0·68-0·79)
African		0·9 (0·89-0·91)	0·94 (0·92-0·97)	0·94 (0·92-0·96)	0·89 (0·86-0·91)	0·86 (0·83-0·89)
Any other Black, African or Caribbean background		0·82 (0·79-0·84)	0·88 (0·82-0·95)	0·87 (0·81-0·94)	0·79 (0·73-0·86)	0·73 (0·65-0·81)
White and Black Caribbean		0·87 (0·85-0·9)	0·91 (0·86-0·98)	0·91 (0·85-0·97)	0·88 (0·82-0·94)	0·83 (0·76-0·9)
White and Black African		0·91 (0·88-0·95)	0·95 (0·87-1·03)	0·95 (0·87-1·03)	0·92 (0·84-1)	0·92 (0·83-1·02)
White and Asian		0·98 (0·94-1·01)	1 (0·92-1·08)	0·99 (0·91-1·07)	0·98 (0·9-1·06)	0·98 (0·9-1·07)
Any other Mixed or multiple ethnic background		0·89 (0·87-0·92)	0·95 (0·89-1·02)	0·95 (0·88-1·02)	0·91 (0·84-0·97)	0·93 (0·86-1·01)
Any other ethnic group		0·91 (0·9-0·93)	0·92 (0·88-0·96)	0·91 (0·88-0·95)	0·86 (0·83-0·9)	0·85 (0·81-0·89)
Unknown		0·97 (0·95-0·98)	0·97 (0·94-1·01)	0·97 (0·94-1)	0·95 (0·91-0·98)	0·93 (0·89-0·97)

Appendix 8. Exploratory analysis: Effect of maternal ethnicity on childhood vaccination, after adjustment for sociodemographic, maternal and birth related factors, and accounting for effect modification by time period where statistically significant (comparing each ethnic group with the White British reference group in the same time period)

These analyses were adjusted for all measured variables in the DAG: IMD, rurality, region, gestational age, mode of birth, maternal age at birth and first-time mother. See Appendix 5 for more details on each of these variables.

A. MMR

**Maternal ethnicity**	**RR (95%CI)**				
	2008-09 to 2010-2011	2011-12 to 2013-14	2014-15 to 2016-17	2017-18 to 2019-20	2020-21
	**Primary course (second birthday)**				
Irish	0·97 (0·94-0·99)	0·96 (0·91-1·01)	0·97 (0·92-1·02)	0·92 (0·87-0·97)	0·93 (0·88-1)
Any other White background	0·98 (0·98-0·99)	0·98 (0·97-0·99)	0·96 (0·94-0·97)	0·91 (0·9-0·93)	0·91 (0·89-0·92)
Indian	1·03 (1·02-1·04)	1·01 (1-1·03)	1 (0·99-1·02)	1·01 (0·99-1·02)	1·01 (0·99-1·03)
Pakistani	1·02 (1·01-1·03)	1·01 (1-1·03)	1 (0·98-1·01)	0·99 (0·97-1·01)	0·99 (0·97-1·01)
Bangladeshi	1·03 (1·02-1·05)	1·03 (1-1·05)	1·02 (1-1·05)	1 (0·97-1·03)	1 (0·97-1·03)
Chinese	0·98 (0·97-1)	0·97 (0·93-1·01)	0·98 (0·94-1·02)	1 (0·96-1·04)	1·01 (0·96-1·05)
Any other Asian background	1·02 (1·01-1·03)	1·01 (0·99-1·03)	1 (0·98-1·02)	1 (0·98-1·02)	1 (0·97-1·02)
Caribbean	0·92 (0·9-0·93)	0·96 (0·92-1)	0·93 (0·89-0·97)	0·79 (0·75-0·83)	0·76 (0·71-0·82)
African	0·96 (0·95-0·97)	0·97 (0·95-0·99)	0·96 (0·94-0·98)	0·9 (0·88-0·92)	0·87 (0·85-0·9)
Any other Black, African or Caribbean background	0·87 (0·85-0·89)	0·88 (0·83-0·94)	0·87 (0·81-0·93)	0·76 (0·71-0·82)	0·73 (0·66-0·81)
White and Black Caribbean	0·93 (0·91-0·95)	0·95 (0·91-1)	0·95 (0·9-1)	0·91 (0·86-0·96)	0·85 (0·79-0·92)
White and Black African	0·94 (0·91-0·97)	0·97 (0·9-1·04)	0·97 (0·9-1·04)	0·93 (0·86-1)	0·93 (0·85-1·01)
White and Asian	1·01 (0·98-1·04)	1·01 (0·95-1·07)	1 (0·94-1·06)	0·98 (0·92-1·04)	0·98 (0·92-1·05)
Any other Mixed or multiple ethnic background	0·94 (0·92-0·97)	0·97 (0·91-1·02)	0·97 (0·92-1·03)	0·93 (0·88-0·98)	0·94 (0·88-1·01)
Any other ethnic group	0·95 (0·94-0·96)	0·96 (0·93-0·99)	0·95 (0·92-0·97)	0·92 (0·89-0·95)	0·89 (0·86-0·93)
Unknown	0·97 (0·96-0·98)	0·97 (0·95-1)	0·97 (0·94-0·99)	0·95 (0·92-0·98)	0·94 (0·9-0·98)
	**Full course (fifth birthday)**				
Irish		0·98 (0·96-1·01)	0·95 (0·89-1)	0·92 (0·86-0·98)	0·93 (0·86-1)
Any other White background		0·96 (0·95-0·97)	0·94 (0·92-0·96)	0·89 (0·87-0·91)	0·87 (0·85-0·89)
Indian		1·02 (1·01-1·03)	1 (0·98-1·02)	0·99 (0·97-1·01)	1·01 (0·98-1·03)
Pakistani		1·01 (1·01-1·02)	1 (0·98-1·02)	0·99 (0·97-1·01)	0·99 (0·97-1·02)
Bangladeshi		1·04 (1·03-1·05)	1·01 (0·99-1·04)	1 (0·97-1·03)	0·96 (0·93-1)
Chinese		0·98 (0·96-1)	0·99 (0·94-1·04)	0·98 (0·93-1·03)	1·01 (0·96-1·07)
Any other Asian background		1·01 (1-1·02)	1 (0·97-1·02)	0·98 (0·96-1)	0·99 (0·96-1·02)
Caribbean		0·93 (0·91-0·95)	0·94 (0·9-0·99)	0·83 (0·79-0·88)	0·72 (0·66-0·79)
African		0·97 (0·96-0·98)	0·97 (0·95-1)	0·93 (0·91-0·95)	0·88 (0·85-0·91)
Any other Black, African or Caribbean background		0·91 (0·89-0·94)	0·89 (0·83-0·96)	0·83 (0·77-0·9)	0·8 (0·72-0·88)
White and Black Caribbean		0·95 (0·93-0·97)	0·96 (0·9-1·01)	0·88 (0·83-0·93)	0·85 (0·79-0·93)
White and Black African		0·97 (0·93-1)	0·94 (0·87-1·02)	0·92 (0·85-1)	0·95 (0·86-1·05)
White and Asian		0·99 (0·96-1·02)	1 (0·93-1·07)	0·98 (0·91-1·05)	0·98 (0·9-1·07)
Any other Mixed or multiple ethnic background		0·97 (0·94-0·99)	0·94 (0·89-1·01)	0·94 (0·88-1)	0·93 (0·86-1·01)
Any other ethnic group		0·93 (0·92-0·95)	0·94 (0·9-0·97)	0·9 (0·86-0·93)	0·87 (0·83-0·92)
Unknown		0·96 (0·95-0·97)	0·96 (0·94-0·99)	0·95 (0·92-0·98)	0·94 (0·89-0·98)

B. 6/5/4-in-1

Maternal ethnicity	RR (95%CI)					
	2007-08	2008-09 to 2010-2011	2011-12 to 2013-14	2014-15 to 2016-17	2017-18 to 2019-20	2020-21
	**Primary course (first birthday)**					
Irish	1 (0·97-1·03)	0·98 (0·92-1·05)	0·98 (0·92-1·04)	0·98 (0·92-1·04)	0·95 (0·89-1·02)	0·98 (0·91-1·05)
Any other White background	1 (0·99-1)	0·99 (0·97-1·01)	0·99 (0·97-1·01)	0·97 (0·95-0·99)	0·94 (0·92-0·96)	0·94 (0·92-0·96)
Indian	1·01 (1-1·02)	1·01 (0·99-1·03)	1·01 (0·99-1·03)	1 (0·98-1·03)	1 (0·97-1·02)	0·99 (0·96-1·02)
Pakistani	1 (0·99-1·01)	1·01 (0·98-1·03)	1 (0·98-1·03)	1 (0·97-1·02)	0·97 (0·95-1)	0·98 (0·95-1·01)
Bangladeshi	0·99 (0·97-1·01)	1·02 (0·98-1·06)	1·01 (0·97-1·05)	1·01 (0·97-1·05)	0·99 (0·95-1·03)	0·99 (0·95-1·04)
Chinese	0·99 (0·96-1·01)	1 (0·94-1·05)	1 (0·95-1·06)	1 (0·95-1·06)	1·01 (0·96-1·07)	0·99 (0·94-1·06)
Any other Asian background	1·01 (1-1·02)	1·01 (0·98-1·03)	1·01 (0·98-1·03)	1·01 (0·98-1·03)	1 (0·97-1·03)	1·01 (0·98-1·04)
Caribbean	0·97 (0·95-0·99)	0·98 (0·94-1·03)	0·97 (0·93-1·02)	0·93 (0·88-0·98)	0·85 (0·81-0·9)	0·77 (0·72-0·83)
African	1·01 (1-1·02)	1·01 (0·99-1·03)	1·01 (0·99-1·03)	1 (0·98-1·02)	0·96 (0·93-0·98)	0·96 (0·93-0·98)
Any other Black, African or Caribbean background	1 (0·98-1·03)	0·99 (0·94-1·05)	0·99 (0·94-1·04)	0·98 (0·93-1·03)	0·92 (0·86-0·98)	0·92 (0·85-0·99)
White and Black Caribbean	0·96 (0·93-0·98)	0·98 (0·92-1·04)	0·96 (0·91-1·02)	0·95 (0·89-1·01)	0·91 (0·85-0·98)	0·89 (0·83-0·97)
White and Black African	0·96 (0·92-1·01)	0·99 (0·9-1·1)	0·98 (0·89-1·09)	0·98 (0·89-1·08)	0·98 (0·88-1·08)	0·97 (0·87-1·08)
White and Asian	1·02 (0·98-1·06)	1 (0·93-1·08)	0·99 (0·92-1·07)	0·98 (0·91-1·06)	0·99 (0·91-1·07)	1 (0·93-1·09)
Any other Mixed or multiple ethnic background	0·94 (0·91-0·98)	0·98 (0·9-1·06)	0·99 (0·91-1·07)	0·97 (0·89-1·05)	0·96 (0·88-1·05)	0·94 (0·86-1·03)
Any other ethnic group	0·96 (0·94-0·98)	0·95 (0·91-0·99)	0·95 (0·91-0·99)	0·94 (0·9-0·98)	0·89 (0·85-0·93)	0·89 (0·85-0·93)
Unknown	0·99 (0·97-1)	0·98 (0·96-1·01)	0·98 (0·95-1·01)	0·96 (0·94-0·99)	0·96 (0·93-0·99)	0·98 (0·94-1·01)
	**Full course (fifth birthday)**					
Irish			0·98 (0·95-1·01)	0·95 (0·88-1·03)	0·9 (0·83-0·97)	0·92 (0·84-1·01)
Any other White background			0·96 (0·95-0·97)	0·94 (0·92-0·96)	0·88 (0·86-0·9)	0·86 (0·83-0·88)
Indian			0·98 (0·97-0·99)	0·99 (0·96-1·02)	0·98 (0·96-1·01)	1·01 (0·98-1·04)
Pakistani			0·98 (0·97-0·99)	0·99 (0·96-1·02)	0·98 (0·96-1·01)	0·99 (0·96-1·02)
Bangladeshi			1·02 (1-1·03)	1·02 (0·98-1·06)	1 (0·97-1·04)	0·99 (0·94-1·04)
Chinese			0·97 (0·94-1)	0·97 (0·91-1·04)	0·98 (0·92-1·04)	0·99 (0·93-1·07)
Any other Asian background			1 (0·98-1·01)	0·99 (0·96-1·03)	0·98 (0·95-1·01)	0·99 (0·95-1·03)
Caribbean			0·94 (0·92-0·96)	0·92 (0·87-0·98)	0·8 (0·75-0·85)	0·7 (0·64-0·78)
African			0·97 (0·96-0·98)	0·98 (0·96-1·01)	0·92 (0·89-0·95)	0·88 (0·85-0·92)
Any other Black, African or Caribbean background			0·93 (0·9-0·96)	0·91 (0·85-0·99)	0·85 (0·78-0·92)	0·82 (0·74-0·92)
White and Black Caribbean			0·95 (0·93-0·98)	0·95 (0·89-1·01)	0·86 (0·81-0·93)	0·85 (0·78-0·93)
White and Black African			0·98 (0·94-1·02)	0·95 (0·86-1·05)	0·91 (0·83-1)	0·95 (0·85-1·06)
White and Asian			0·99 (0·95-1·04)	1 (0·91-1·1)	0·96 (0·87-1·05)	0·97 (0·87-1·08)
Any other Mixed or multiple ethnic background			0·95 (0·91-0·98)	0·95 (0·87-1·03)	0·93 (0·86-1·01)	0·91 (0·82-1)
Any other ethnic group			0·94 (0·92-0·96)	0·92 (0·88-0·97)	0·89 (0·84-0·93)	0·84 (0·8-0·89)
Unknown			0·96 (0·95-0·98)	0·96 (0·92-1)	0·94 (0·91-0·98)	0·94 (0·89-0·99)

C. MenC and Hib/MenC

Maternal ethnicity	RR (95%CI)				
	2008-09 to 2010-2011	2011-12 to 2013-14	2014-15 to 2016-17	2017-18 to 2019-20	2020-21
	**Primary course MenC (first birthday)**				
Irish	0·99 (0·98-1)				
Any other White background	0·98 (0·98-0·99)				
Indian	1 (1-1)				
Pakistani	0·99 (0·99-1)				
Bangladeshi	1·01 (1-1·01)				
Chinese	0·99 (0·99-1)				
Any other Asian background	1 (1-1)				
Caribbean	0·97 (0·96-0·97)				
African	1 (1-1·01)				
Any other Black, African or Caribbean background	0·99 (0·98-1)				
White and Black Caribbean	0·97 (0·96-0·98)				
White and Black African	0·98 (0·97-0·99)				
White and Asian	0·99 (0·98-1)				
Any other Mixed or multiple ethnic background	0·97 (0·97-0·98)				
Any other ethnic group	0·94 (0·94-0·95)				
Unknown	0·98 (0·97-0·98)				
	**Hib/MenC (second birthday)**				
Irish	0·98 (0·95-1)	0·97 (0·92-1·02)	0·97 (0·92-1·02)	0·93 (0·88-0·98)	0·93 (0·88-1)
Any other White background	0·98 (0·97-0·99)	0·98 (0·96-0·99)	0·96 (0·95-0·97)	0·92 (0·9-0·93)	0·91 (0·89-0·93)
Indian	1·01 (1·01-1·02)	1 (0·99-1·02)	1 (0·98-1·02)	1·01 (0·99-1·02)	1·01 (1-1·03)
Pakistani	1·02 (1·01-1·03)	1·01 (1-1·03)	1 (0·98-1·02)	0·99 (0·97-1·01)	0·99 (0·97-1·01)
Bangladeshi	1·03 (1·01-1·04)	1·03 (1-1·05)	1·03 (1-1·05)	1 (0·97-1·03)	1 (0·97-1·03)
Chinese	0·94 (0·92-0·97)	0·94 (0·9-0·99)	0·96 (0·92-1·01)	1 (0·95-1·04)	1 (0·95-1·06)
Any other Asian background	1·01 (1-1·02)	1·01 (0·99-1·03)	1 (0·98-1·02)	1 (0·98-1·02)	1 (0·98-1·03)
Caribbean	0·94 (0·92-0·96)	0·97 (0·93-1·01)	0·93 (0·89-0·97)	0·79 (0·75-0·83)	0·77 (0·72-0·83)
African	0·98 (0·97-0·98)	0·98 (0·96-1)	0·97 (0·95-0·99)	0·92 (0·9-0·94)	0·88 (0·86-0·91)
Any other Black, African or Caribbean background	0·93 (0·91-0·95)	0·94 (0·89-0·99)	0·91 (0·86-0·96)	0·83 (0·78-0·88)	0·76 (0·69-0·83)
White and Black Caribbean	0·95 (0·93-0·97)	0·97 (0·92-1·01)	0·95 (0·91-1)	0·91 (0·86-0·96)	0·86 (0·8-0·92)
White and Black African	0·99 (0·96-1·01)	0·98 (0·92-1·04)	0·98 (0·93-1·05)	0·94 (0·88-1·01)	0·93 (0·86-1·01)
White and Asian	1·01 (0·98-1·03)	1 (0·94-1·06)	1·01 (0·95-1·07)	0·98 (0·92-1·04)	0·98 (0·92-1·06)
Any other Mixed or multiple ethnic background	0·96 (0·93-0·98)	0·97 (0·92-1·03)	0·97 (0·92-1·03)	0·92 (0·87-0·97)	0·94 (0·88-1·01)
Any other ethnic group	0·95 (0·93-0·96)	0·95 (0·92-0·98)	0·94 (0·91-0·97)	0·9 (0·87-0·93)	0·89 (0·85-0·92)
Unknown	0·93 (0·92-0·95)	0·97 (0·94-1)	0·96 (0·94-0·99)	0·96 (0·93-0·99)	0·94 (0·9-0·98)

D. MenB

Maternal ethnicity	RR (95%CI)
	**Primary course (first birthday)**
Irish	0·98 (0·97-0·99)
Any other White background	0·94 (0·94-0·95)
Indian	1 (0·99-1)
Pakistani	0·98 (0·98-0·99)
Bangladeshi	1 (0·99-1·01)
Chinese	1 (1-1·01)
Any other Asian background	1 (1-1·01)
Caribbean	0·83 (0·81-0·85)
African	0·97 (0·97-0·98)
Any other Black, African or Caribbean background	0·94 (0·93-0·95)
White and Black Caribbean	0·92 (0·91-0·93)
White and Black African	0·97 (0·95-0·98)
White and Asian	0·98 (0·97-1)
Any other Mixed or multiple ethnic background	0·95 (0·94-0·96)
Any other ethnic group	0·9 (0·89-0·91)
Unknown	0·96 (0·95-0·97)
	**Full course (second birthday)**
Irish	0·92 (0·9-0·94)
Any other White background	0·91 (0·91-0·92)
Indian	1 (0·99-1·01)
Pakistani	0·99 (0·98-1)
Bangladeshi	1·02 (1·01-1·03)
Chinese	1 (0·99-1·01)
Any other Asian background	1·01 (1-1·02)
Caribbean	0·77 (0·75-0·79)
African	0·91 (0·9-0·92)
Any other Black, African or Caribbean background	0·8 (0·77-0·82)
White and Black Caribbean	0·89 (0·87-0·92)
White and Black African	0·93 (0·9-0·95)
White and Asian	0·98 (0·96-1)
Any other Mixed or multiple ethnic background	0·94 (0·92-0·95)
Any other ethnic group	0·87 (0·86-0·89)
Unknown	0·95 (0·93-0·96)

E. Rotavirus

Maternal ethnicity	RR (95%CI)
	Primary course (first birthday)
Irish	0·97 (0·95-0·98)
Any other White background	0·94 (0·94-0·95)
Indian	1 (1-1·01)
Pakistani	0·96 (0·96-0·97)
Bangladeshi	0·98 (0·97-0·99)
Chinese	1·01 (1-1·02)
Any other Asian background	1 (0·99-1)
Caribbean	0·86 (0·85-0·87)
African	0·97 (0·97-0·98)
Any other Black, African or Caribbean background	0·93 (0·92-0·95)
White and Black Caribbean	0·92 (0·91-0·94)
White and Black African	0·96 (0·94-0·97)
White and Asian	0·98 (0·96-0·99)
Any other Mixed or multiple ethnic background	0·94 (0·93-0·96)
Any other ethnic group	0·88 (0·87-0·89)
Unknown	0·96 (0·95-0·97)

F. Pneumococcal

Maternal ethnicity	RR (95%CI)					
	2007-08	2008-09 to 2010-2011	2011-12 to 2013-14	2014-15 to 2016-17	2017-18 to 2019-20	2020-21
	**Primary course (first birthday)**					
Irish	1·01 (0·96-1·05)	0·99 (0·91-1·07)	0·98 (0·9-1·06)	0·98 (0·9-1·07)	0·97 (0·89-1·06)	0·99 (0·9-1·08)
Any other White background	1 (0·98-1·01)	0·99 (0·96-1·02)	0·99 (0·96-1·02)	0·97 (0·94-0·99)	0·95 (0·92-0·97)	0·94 (0·92-0·97)
Indian	0·99 (0·98-1·01)	1 (0·97-1·04)	1·01 (0·97-1·04)	1 (0·97-1·04)	1·01 (0·97-1·04)	1·01 (0·97-1·04)
Pakistani	0·97 (0·96-0·99)	1 (0·97-1·04)	1 (0·96-1·04)	1 (0·96-1·04)	0·99 (0·95-1·03)	0·98 (0·94-1·02)
Bangladeshi	0·98 (0·95-1·01)	1·02 (0·96-1·07)	1·01 (0·96-1·07)	1·01 (0·96-1·07)	1 (0·95-1·05)	0·98 (0·92-1·04)
Chinese	0·99 (0·96-1·03)	0·99 (0·92-1·07)	1 (0·93-1·07)	1 (0·93-1·08)	1·01 (0·94-1·09)	1 (0·93-1·07)
Any other Asian background	1 (0·98-1·02)	1 (0·97-1·04)	1·01 (0·97-1·05)	1 (0·97-1·04)	1 (0·97-1·04)	1 (0·96-1·04)
Caribbean	0·92 (0·89-0·95)	0·98 (0·92-1·05)	0·98 (0·91-1·05)	0·93 (0·87-1)	0·84 (0·78-0·91)	0·79 (0·73-0·87)
African	0·97 (0·96-0·99)	1 (0·97-1·04)	1·01 (0·98-1·04)	1·01 (0·97-1·04)	0·98 (0·95-1·01)	0·96 (0·93-1)
Any other Black, African or Caribbean background	0·95 (0·91-0·99)	0·99 (0·91-1·08)	0·99 (0·91-1·08)	0·99 (0·91-1·07)	0·94 (0·87-1·02)	0·94 (0·85-1·03)
White and Black Caribbean	0·93 (0·89-0·96)	0·97 (0·89-1·05)	0·96 (0·89-1·05)	0·95 (0·87-1·03)	0·93 (0·85-1·01)	0·9 (0·82-0·99)
White and Black African	0·93 (0·87-1)	0·99 (0·86-1·13)	0·98 (0·86-1·12)	0·97 (0·85-1·11)	0·97 (0·85-1·11)	0·98 (0·85-1·13)
White and Asian	0·95 (0·89-1·02)	1 (0·86-1·15)	0·99 (0·86-1·14)	0·99 (0·85-1·14)	0·99 (0·85-1·14)	1 (0·86-1·15)
Any other Mixed or multiple ethnic background	0·92 (0·87-0·97)	0·97 (0·87-1·09)	0·99 (0·89-1·1)	0·97 (0·87-1·08)	0·96 (0·86-1·07)	0·94 (0·84-1·06)
Any other ethnic group	0·95 (0·93-0·97)	0·95 (0·9-1)	0·96 (0·91-1·01)	0·94 (0·89-0·99)	0·91 (0·86-0·96)	0·9 (0·85-0·96)
Unknown	0·99 (0·97-1)	0·99 (0·95-1·02)	0·99 (0·95-1·02)	0·96 (0·93-1)	0·96 (0·92-0·99)	0·97 (0·94-1·02)
	**Full course (second birthday)**					
Irish		0·95 (0·92-0·98)	0·96 (0·9-1·03)	0·96 (0·9-1·03)	0·93 (0·87-0·99)	0·93 (0·86-1·01)
Any other White background		0·98 (0·97-0·98)	0·98 (0·96-1)	0·96 (0·94-0·97)	0·91 (0·9-0·93)	0·91 (0·89-0·93)
Indian		1·01 (1-1·02)	1·01 (0·99-1·03)	1 (0·98-1·03)	1·01 (0·99-1·03)	1·02 (0·99-1·04)
Pakistani		1 (0·99-1·01)	1·01 (0·98-1·03)	1 (0·97-1·02)	0·99 (0·97-1·01)	0·99 (0·96-1·02)
Bangladeshi		1·02 (1-1·03)	1·03 (0·99-1·06)	1·03 (1-1·06)	1·01 (0·97-1·04)	1 (0·96-1·05)
Chinese		0·96 (0·94-0·99)	0·96 (0·91-1·02)	0·97 (0·92-1·02)	1 (0·95-1·05)	1·01 (0·95-1·07)
Any other Asian background		1·02 (1-1·03)	1·01 (0·99-1·04)	1 (0·97-1·02)	1 (0·98-1·03)	1·01 (0·98-1·04)
Caribbean		0·89 (0·87-0·91)	0·96 (0·91-1·01)	0·92 (0·88-0·97)	0·77 (0·72-0·81)	0·76 (0·7-0·83)
African		0·94 (0·93-0·95)	0·98 (0·96-1·01)	0·98 (0·95-1)	0·92 (0·9-0·95)	0·89 (0·86-0·92)
Any other Black, African or Caribbean background		0·85 (0·82-0·88)	0·92 (0·86-0·99)	0·91 (0·84-0·97)	0·82 (0·76-0·89)	0·75 (0·68-0·84)
White and Black Caribbean		0·89 (0·87-0·92)	0·94 (0·88-1)	0·94 (0·88-1)	0·9 (0·84-0·96)	0·85 (0·78-0·92)
White and Black African		0·93 (0·9-0·97)	0·97 (0·89-1·06)	0·97 (0·89-1·05)	0·94 (0·86-1·02)	0·94 (0·85-1·03)
White and Asian		0·99 (0·95-1·02)	1 (0·93-1·09)	0·99 (0·92-1·08)	0·98 (0·91-1·07)	0·99 (0·9-1·08)
Any other Mixed or multiple ethnic background		0·92 (0·89-0·95)	0·97 (0·9-1·04)	0·96 (0·9-1·03)	0·92 (0·86-0·99)	0·95 (0·87-1·02)
Any other ethnic group		0·93 (0·92-0·95)	0·94 (0·9-0·97)	0·93 (0·9-0·97)	0·88 (0·85-0·92)	0·87 (0·83-0·91)
Unknown		0·96 (0·95-0·97)	0·97 (0·94-1)	0·96 (0·93-0·99)	0·95 (0·91-0·98)	0·93 (0·89-0·97)

References

1. Inns T, Fleming KM, Iturriza-Gomara M, Hungerford D. Paediatric rotavirus vaccination, coeliac disease and type 1 diabetes in children: a population-based cohort study. BMC Medicine. 2021;19(1):147.
2. Walker JL, Andrews NJ, Atchison CJ, Collins S, Allen DJ, Ramsay ME, et al. Effectiveness of oral rotavirus vaccination in England against rotavirus-confirmed and all-cause acute gastroenteritis. Vaccine: X. 2019;1:100005.
3. Davidson J, Banerjee A, Mathur R, Ramsay M, Smeeth L, Walker J, et al. Ethnic differences in the incidence of clinically diagnosed influenza: an England population-based cohort study 2008-2018. Wellcome Open Research. 2021;6(49).
4. Zhang CX, Boukari Y, Pathak N, Mathur R, Katikireddi SV, Patel P, et al. Migrants' primary care utilisation before and during the COVID-19 pandemic in England: An interrupted time series analysis. The Lancet Regional Health – Europe. 2022;20.
5. Shiekh SI, Harley M, Ghosh RE, Ashworth M, Myles P, Booth HP, et al. Completeness, agreement, and representativeness of ethnicity recording in the United Kingdom's Clinical Practice Research Datalink (CPRD) and linked Hospital Episode Statistics (HES). Population Health Metrics. 2023;21(1):3.
6. NHS Digital. NHS Data Migration Pack April 2020. 2020.
7. Clinical Practice Research Datalink. Hospital Episode Statistics (HES) Admitted Patient Care and CPRD primary care data Documentation (set 22/January 2022, Version 2.8). 2021.
8. Public Health England. Outputs by ethnic group in PHE's COVID-19 Health Inequalities Monitoring for England (CHIME) tool. Available from: https://fingertips.phe.org.uk/documents/Outputs%20by%20ethnic%20group%20in%20CHIME.pdf.
9. Scobie S, Spencer J, Raleigh V. Ethnicity coding in English health service datasets 2021. Available from: https://www.nuffieldtrust.org.uk/research/ethnicity-coding-in-english-health-service-datasets.
10. Mathur R, Bhaskaran K, Chaturvedi N, Leon DA, vanStaa T, Grundy E, et al. Completeness and usability of ethnicity data in UK-based primary care and hospital databases. Journal of Public Health. 2014;36(4):684-92.
11. Mathur R. Ethnic inequalities in health and use of healthcare in the UK: how computerised health records can contribute substantively to the knowledge base London School of Hygiene & Tropical Medicine; 2015.
12. Williams DR, Lawrence JA, Davis BA, Vu C. Understanding how discrimination can affect health. Health Services Research. 2019;54(S2):1374-88.
13. Lymperopoulou K, Finney N. Socio-spatial factors associated with ethnic inequalities in districts of England and Wales, 2001–2011. Urban Studies. 2016;54(11):2540-60.
14. Slopen N, Heard-Garris N. Structural Racism and Pediatric Health—A Call for Research to Confront the Origins of Racial Disparities in Health. JAMA Pediatrics. 2022;176(1):13-5.
15. Andersen RM. Revisiting the Behavioral Model and Access to Medical Care : Does it Matter ? Journal of Health and Social Behavior1995. p. 1-10.
16. Levesque JF, Harris MF, Russell G. Patient-centred access to health care: Conceptualising access at the interface of health systems and populations. International Journal for Equity in Health: BioMed Central; 2013. p. 18.
17. Boyle EM, Poulsen G, Field DJ, Kurinczuk JJ, Wolke D, Alfirevic Z, et al. Effects of gestational age at birth on health outcomes at 3 and 5 years of age: population based cohort study. BMJ. 2012;344:e896.
18. Katikireddi SV, Lal S, Carrol ED, Niedzwiedz CL, Khunti K, Dundas R, et al. Unequal impact of the COVID-19 crisis on minority ethnic groups: a framework for understanding and addressing inequalities. Journal of Epidemiology and Community Health. 2021;75:970–4.
19. Mahmud SM, Xu L, Hall LL, Puckrein G, Thommes E, Loiacono MM, et al. Effect of race and ethnicity on influenza vaccine uptake among older US Medicare beneficiaries: a record-linkage cohort study. The Lancet Healthy Longevity. 2021;2(3):e143-e53.
20. Devakumar D, Selvarajah S, Abubakar I, Kim S-S, McKee M, Sabharwal NS, et al. Racism, xenophobia, discrimination, and the determination of health. The Lancet. 2022;400(10368):2097-108.
21. Routen A, Akbari A, Banerjee A, Katikireddi SV, Mathur R, McKee M, et al. Strategies to record and use ethnicity information in routine health data. Nature Medicine. 2022.
22. Tennant PWG, Murray EJ, Arnold KF, Berrie L, Fox MP, Gadd SC, et al. Use of directed acyclic graphs (DAGs) to identify confounders in applied health research: review and recommendations. International Journal of Epidemiology. 2021;50(2):620-32.
23. Ministry of Housing CLG. English indices of deprivation 2019 2019. Available from: https://www.gov.uk/government/statistics/english-indices-of-deprivation-2019.
